# The Effectiveness of Traditional Chinese Medicine (TCM) as an Adjunct Treatment on Stable COPD Patients: A Systematic Review and Meta-Analysis

**DOI:** 10.1155/2021/5550332

**Published:** 2021-06-04

**Authors:** K. H. Chan, Y. Y. S. Tsoi, M. McCall

**Affiliations:** ^1^Department for Continuing Education, The University of Oxford, England, UK; ^2^Department of Primary Care Health Sciences, The University of Oxford, England, UK; ^3^Independent Researcher Hong Kong China, The University of Oxford, Hong Kong, China

## Abstract

**Background:**

Traditional Chinese medicine (TCM), including Chinese herbal medicine (CHM) and acupuncture, exhibits beneficial effects on stable chronic obstructive pulmonary disease (COPD) such as improving lung function and reducing exacerbation. Previous research studies have examined either CHM or acupuncture alone, which are not the usual practice in TCM clinic setting. We conduct a systematic review for evaluating the clinical effectiveness and safety of TCM by combining CHM and acupuncture.

**Methods:**

Databases are searched from inception to November 2019. Randomized controlled trials examining either acupuncture or CHM on stable COPD are included. Primary outcomes include lung functions, exacerbations, and COPD assessment test. Secondary outcomes include quality of life, TCM syndrome score and effective rate, and 6-minute walk distance. Two independent reviewers extract data and assess the quality of evidence and generate meta-analysis and risk of bias by STATA. This protocol follows the Preferred Reporting Items for Systematic Review and Meta-Analysis Protocols (PRISMA-P) guidelines.

**Results:**

100 randomized controlled trials (8291 participants) were included to compare add-on Chinese medicine treatment with conventional treatment (CT). Combining CHM with CT improves FEV_1_ (MD: 0.18, 95% CI: 0.08, 0.28), exacerbation rate (MD: −0.29, 95% CI: −0.61, 0.03), COPD assessment test (MD: −2.16, 95% CI: −3.44, -0.88), TCM syndrome score (MD: −3.96, 95% CI: −5.41, −2.51) and effective rate (RR: 0.89, 95% CI: 0.80, 0.93), and 6-minute walk test (MD: 37.81, 95% CI: 20.90, 54.73). No serious adverse events were reported. Risk of bias: *low* to *unclear*.

**Conclusions:**

This review identifies sufficient moderate-to-low-quality evidence to suggest TCM as an adjunct treatment for stable COPD patients. Though heterogeneity was low among studies, the results were limited and the quality of evidence was low or very low based on small sample sizes and risk of bias. Future studies with larger sample sizes are warranted. The trial is registered with CRD42019161324.

## 1. Introduction

Chronic obstructive pulmonary disease (COPD) is a common, treatable, and preventable disease, which is characterized by chronic respiratory symptoms and airflow limitation owing to airway and/or alveolar abnormalities caused by persistent exposure to noxious gases or molecules. The major known pathogenesis of COPD is a complex mixture of small airway disease, parenchymal destruction, and chronic airway and/or systemic inflammation.

COPD is an important cause of chronic morbidity and mortality in the world, which ranks the fourth in the leading cause of death and is projected to be the third by 2020 [1, 2]. It is a common, preventable, and treatable disease but poses an economic burden on the society. COPD patients are usually characterized by persistent respiratory symptoms and airflow limitation. Occasionally, they may have acute exacerbation induced by respiratory infection and increase the hospitalization and readmission rate.

Current COPD prevalence data show significant differences among countries, probably because of different diagnostic criteria, survey techniques, and analytical methods [3]. The Burden of Obstructive Lung Diseases (BOLD) program has reported the prevalence and risk factors for COPD in people aged ≥40 in more than 29 countries and found that COPD is more common in men than women [4, 5]. Up to now, there are around three million deaths per year [6]. The prevalence of COPD is predicted to rise in the coming 30 years, and by 2030, there might be over 4.5 million deaths per year from COPD and comorbidities [7, 8].

Diagnosis of COPD is primarily by spirometry which measures the patient's airflow limitation. It is the most widely accepted, easily available, and reproducible test of lung function. A ratio of postbronchodilator forced expiratory volume in first second (FEV_1_)/forced vital capacity (FVC) <0.70 confirms the presence of persistent airflow limitation [9]. Main symptoms include dyspnea, chronic cough, chronic sputum production, wheezing, and chest tightness. But the severity of airflow limitation is weakly correlated with symptoms in clinical context [10], and spirometry itself has a relatively low specificity [11]. So other symptom assessments are required to categorize COPD patients, which commonly include the Modified British Medical Research Council (mMRC) Questionnaire [12] and COPD Assessment Test (CAT^TM^) [13–15].

COPD patients may suffer acute worsening of respiratory symptoms that lead to additional therapy, namely, acute exacerbations [16–19]. There are three classifications of exacerbations: mild (short-acting bronchodilators (SABDs) only), moderate (SABDs plus antibiotics and/or oral corticosteroids), and severe (hospitalization or visiting emergency room). The best indicator of frequent exacerbations (defined as two or more exacerbations per annum) is a history of earlier treated events [20]. Apart from these tests, physical exercise measurements, such as paced shuttle walk test and the unpaced 6-minute walk test, are also suggested for monitoring patient health status and predicting prognosis [21–23].

For stable COPD, the goals of pharmacological therapy are to reduce symptoms, reduce the frequency and severity of exacerbations, and improve health status and exercise tolerance. Apart from smoking cessation and vaccinations, there are two major classes of medications: bronchodilators and anti-inflammatory drugs. Bronchodilators can increase FEV_1_ and/or modify other spirometric values and are usually prescribed regularly to prevent or reduce symptoms. Commonly used bronchodilators include short-acting and long-acting beta_2_-agonists (SABA and LABA, respectively) and short-acting and long-acting anticholinergics (SAMA and LAMA, respectively) [24–27].

Traditional Chinese medicine (TCM) has been using to treat symptoms similar to those in COPD, for instance, cough, sputum, or shortness of breath and has shown beneficial effects for over hundreds of decades. However, there is no such a disease term as COPD in TCM. Instead, COPD patients are classified as having “Fei Zhang” with reference to TCM theory [28]. In a normal TCM clinical setting, either Chinese herbal medicine, acupuncture, or the combination of both is used to relieve COPD symptoms and improve lung functions and/or exercise tolerance [29–32].

TCM is very different from contemporary medicine in both diagnosis and treatment methods. Commonly used TCM treatments include herbal medicinal formula, acupuncture, moxibustion, Tuina, or the combination of them. In a daily TCM healthcare setting, patients with COPD symptoms are often given a set of treatments such as acupuncture/moxibustion, or acupuncture/medicinal formula. Most RCTs for TCM treatments were conducted only on several acupoints, or a single herb or formulae, which is not similar to the usual TCM practice. This study aims to examine the effectiveness and adverse effects of adding TCM treatments on western medicine in stable COPD, to synthesize the best available data towards recommendations of optimal treatment.

The primary objective of this study is to measure the effectiveness of TCM as an adjunct treatment on stable COPD patients in any setting and the adverse events associated with its use in clinical trials measured by lung function and exacerbation rate. The secondary objective of this study is to compare the efficacy of either herbal medicine, acupuncture, or the combination of both on treating stable COPD patients reflected by TCM syndrome score and health status.

  Population: patients with stable COPD aged >18 years old, of any sex, education, and socioeconomic status
  Interventions: add-on TCM treatment, either herbal medicine, acupuncture, or the combination of both, on conventional medicine
  Controls/comparators: mainstream pharmacotherapy for managing stable COPD
  Outcomes: lung functions as measured by FEV_1_ using spirometry, exacerbation rate, 6-minute walk test, and health-related quality of life (QoL)
  Study design: double-blind, randomized controlled clinical trials

## 2. Methods

This systematic review was prepared with reference to the Preferred Reporting Items for Systematic Review and Meta-Analysis Protocols (PRISMA-P) [33] and the Cochrane Handbook for Systematic Reviews of Interventions [32] and registered on the international prospective register of systematic review (PROSPERO) on 10.12.2019 (registration number: CRD42019161324). Research protocol and supplementary information are listed in Appendices 1–5.

### 2.1. Inclusion and Exclusion Criteria for Studies

#### 2.1.1. Types of Included Studies

Any randomized controlled trials (RCT) with double-blind assessment of patient-reported outcomes, of which both patients and assessors were blind to the treatments given, were included. RCTs published in a peer-reviewed journal with full text were requested, and unpublished clinical trials with online results available were included.

#### 2.1.2. Types of Excluded Studies

Abstracts alone, nonrandomized trials, case reports, cohort studies, case-control studies, cross-sectional studies, retrospective surveys or chart reviews, editorials, commentaries, and clinical observations were excluded from this systematic review. Other systematic reviews were not included, but the reference lists of similar were searched.

#### 2.1.3. Types of Included Participants

Our search was designed to include (1) patients who were 18 years old or above, regardless of sex, education, race, and socioeconomic status, and (2) patients who were diagnosed with stable COPD according to the diagnostic criteria from the Global Initiative for Chronic Obstructive Lung Disease (GOLD) [6]. Stable COPD patients were defined as patients having mild cough, expectoration, and dyspnea.

#### 2.1.4. Types of Excluded Participants

We excluded patients with other diseases such as asthma, tuberculosis, bronchiolitis, congestive heart failure, or other severe complications because we only wanted to examine the efficacy of TCM on stable COPD.

#### 2.1.5. Types of Interventions

We included any herbal drugs, extracted active ingredients, or formula administered orally, which could be either in a form of TCM granules or boiled soup, and compared to no treatment, placebo, or any active comparator plus conventional medicine. We also included any acupuncture treatment, or dry needling, using any acupoint combinations, and compared to no treatment, placebo, or any active comparator plus conventional medicine. Studies in any healthcare and any global setting were included. Interventions either alone or in combination with each other were included.

#### 2.1.6. Types of Outcome Measures

*Primary Outcomes*. We included the following items as primary outcomes: (1) lung functions by measuring the change in FEV_1_ [35]; (2) exacerbations defined as time-to-first exacerbation or exacerbation rate [6]; (3) COPD assessment test [36]; and (4) adverse events of any cause.

*Secondary Outcomes*. As an assessment of COPD patients' quality of life, we included quality of life such as sleep patterns, mood, and mental health and physical exercise regime on a validated scale; (2) TCM syndrome score and effective rate [37]; and (3) 6-minute walk distance [38] as secondary outcomes.

*Search Strategy*. The lead author (KH) designed the search strategy and carry out the searches. A broad search strategy was used to cover all Chinese herbal medicine and acupuncture RCTs to include as many relevant and potentially included trials as possible, from studies inception to November 2019.

*Electronic Searches*. The following databases were searched mainly in English and Chinese languages and filtered for humans:

1. PubMed
2. MEDLINE
3. EMBASE
4. Cochrane Central Register of Controlled Trials (CENTRAL)
5. Chinese National Knowledge Infrastructure (CNKI)
6. WANFANG Database
7. Chinese Scientific and Technological Periodical Database (VIP)
8. Chinese Biomedical Database (CBM)
9. Cochrane Library Database

The search strategies were tailor-made to each database with a combination of text words and medical subject headings (MeSH), or an equivalent, and search terms are listed in Table 1.

Moreover, the following online registries were searched in English and Chinese language and filtered for humans:

1. ClinicalTrials.gov
2. The metaRegister of controlled trials (mRCT)
3. The World Health Organization (WHO) International Clinical Trials Registry Platform (ICTRP)

*Searching Other Resources*. Bibliographies and reference lists of related publications which match the eligibility criteria were hand searched, such that we did not miss any important references during the selection process.

### 2.2. Data Collection and Analysis

#### 2.2.1. Data Extraction and Management

Two reviewers (KH and YYS) independently extracted study information and outcome data using a standardized data extraction table for RCTs only [39] that includes title, first author, publication year, country, sample size, age and sex of participants, intervention, treatment duration, follow-up period, outcomes, and adverse events. Extracted data were cross-checked and entered into STATA (version 16). Any disagreements about extracted data were adjudicated by the third reviewer (MM) and were resolved by discussion and consensus.

#### 2.2.2. Risk of Bias Assessment

Two authors (KH and YYS) independently assessed the risk of bias for each record using the Cochrane Risk of Bias Tool as reported in the Cochrane Handbook for Systematic Reviews of Interventions [32].

A risk of bias table was included as part of each characteristic of included studies table. When facing disagreements about the risk of bias, a third reviewer (MM) adjudicated and disagreements were resolved by discussion. The risk of bias was assessed at the individual study level and the risk of bias was also considered when assessing Grading of Recommendations, Assessment, Development, and Evaluation system (GRADE) [40].

These seven domains were assessed for each included study as outlined by the Cochrane Handbook for Systematic Reviews of Interventions [32]:

1. Random sequence generation (examine potential selection bias): studies were assessed for the methods used to generate the allocation sequence
2. Allocation concealment (examine potential selection bias): studies were assessed for the methods used to conceal allocation to interventions before the study starts
3. Blinding of participants and personnel (examine potential performance bias): studies were assessed for methods used to blind the participants and personnel from knowing which intervention a participant would receive
4. Blinding of outcome assessment (examine potential detection bias): studies were assessed for methods used to blind the outcome assessors from knowing which intervention a participant would receive
5. Incomplete outcome data (examine potential attrition bias): studies were assessed for the nature, number, and handling of incomplete outcome data
6. Selective reporting (examine potential reporting bias): studies were assessed whether all planned outcomes were reported in the results
7. Other bias: studies were assessed for any additional sources of bias as low, unclear, or high and provided rationale

#### 2.2.3. Assessment of Heterogeneity

To evaluate clinical heterogeneity, only studies with similar conditions and treatments were compared to get a clinically useful result [32]. Statistical heterogeneity was assessed visually [41] with the *I*^2^ statistic and *p* value. If *I*^2^ was larger than 50%, possible reasons were discussed [32].

#### 2.2.4. Data Synthesis

The meta-analysis was conducted on extracted data using STATA (version 16) using a random-effects model. Binary data were expressed as risk ratio with 95% confidence intervals (CIs) and were analyzed by Mantel–Haenszel method. For continuous variables, mean difference (MD) with 95% CIs are calculated. Heterogeneity was examined by *I*^2^ tests.

*Quality of Evidence*. GRADE was used to assess the quality of evidence related to each outcome measure and to provide recommendations for clinical practice [32, 40]. A GRADE rating was assigned for each primary and secondary outcome using the four key levels: high, moderate, low, or very low quality, with reasons provided to upgrade or downgrade [40]. Under certain circumstances, the overall GRADE rating might require adjustment. For instance, a study reported very small sample sizes and results were at risk of being down to play of chance [42]. On the other hand, if no data were reported for an outcome, the term “no evidence” or “lack of evidence” could imply there were data and that the results might show no evidence of effect.

#### 2.2.5. Subgroup Analysis

Subgroup analyses were performed to assess factors such as different TCM dosage, forms, duration of treatment, acupoints used, and measurements of results to see whether they have any impact on the effect estimate. Sensitivity analysis was conducted to examine heterogeneity. The effect of methodological quality, sample size, or missing data was also considered. Analysis was repeated after removing methodologically low-quality studies.

#### 2.2.6. Publication Bias

If more than ten studies were selected, the Egger regression test was used to assess any possible publication bias [43].

#### 2.2.7. Ethical Considerations

There were no ethical issues or approvals needed for this type of study as it used aggregate data already anonymized.

## 3. Results

### 3.1. Study Identification

**Figure 1:**
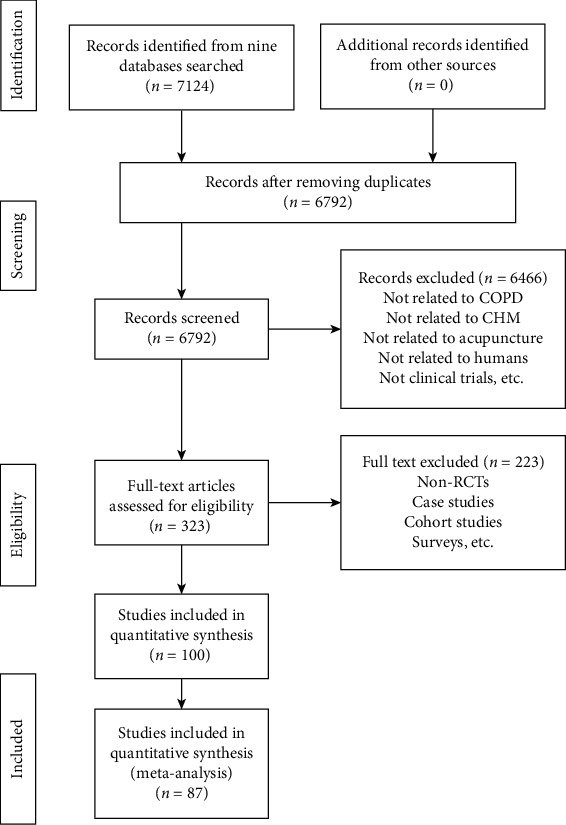
PRISMA study flowchart of search results.

The PRISMA study flowchart of search results is shown in Figure 1.

Updated on 1 March 2020, a total of 7124 records are identified from databases and 0 records are found from other sources. After removing duplicates, 6792 titles and abstracts are screened and 323 full-text articles are obtained. Among them, 100 articles are included and 223 are excluded with reasons provided in Appendix 7. No studies are ongoing or under assessment.

### 3.2. Description of Included Studies

Table 2 summarizes the basic characteristics of 100 included studies. We report sample sizes, ages, course of the disease, and gender for control and intervention groups. Types of Chinese medicine, treatment duration, baseline difference, and quality control are also listed.

#### 3.2.1. Design

All studies are randomized, double-blind, and controlled clinical trials and report primary and secondary outcomes.

#### 3.2.2. Sample Size

Sample sizes range from 15 participants per arm [44] to 83 participants per arm [45]. Ages range from 40.32 ± 3.12 (mean ± SE) [46] to 71.2 ± 5.7 [47] in the control group and from 40.65 ± 3.08 [46] to 72.35 ± 4.77 [47] in the intervention group. Ten studies do not report the mean age [48–57].

#### 3.2.3. Participants

All studies recruit participants according to the GOLD guidelines [6], and all participants are in stable phase with stages between II and IV. The course of disease (in years) ranges from 3.03 ± 0.38 (mean ± SE) [58] to 33.57 ± 10.97 in the control group [28] and from 3.05 ± 0.37 [58] to 33.26 ± 9.41 [28] in the intervention group. Thirty studies do not report the course of years shown in Table 2.

#### 3.2.4. Setting and Location

All studies are single-centered trials and based in hospital settings. The location of studies scatters across different provinces in China.

#### 3.2.5. Interventions

*Comparison Arms*. There is no acupuncture plus conventional medicine versus conventional treatment (CT) identified. Ninety-nine studies compare one Chinese herbal medicine (CHM) formulae plus CT with conventional medicine. One study has three arms: conventional medicine, CHM decoction plus CT, and CHM powder plus CT [48].

*Types of Chinese Medicine*. One hundred different CHM formulae are used and detailed compositions and dosages of each formula are shown in Appendix 6. Conventional medicine is prescribed with reference to GOLD guidelines.

*Duration of Treatment*. The duration of treatment differs across studies, which ranges from 1 week [59] to 42 weeks [60]. The mean duration of treatment is 14 weeks. Two studies do not report the duration of treatment [61, 62].

#### 3.2.6. Outcomes

Table 3 summarizes the availability of outcome measures reported. Forty studies report a change in FEV_1_ (mean ± SE). Thirteen studies report exacerbation rate (mean ± SE) as a continuous outcome at the study endpoint. Twenty studies report COPD assessment test (mean ± SE). Twenty-five studies report 6-minute walk test (mean ± SE). Thirty-five studies report TCM syndrome score (mean ± SE) and sixty-two studies report TCM effective rate as dichotomous outcome. For quality of life, seventeen studies report in different QoL scales (mean ± SE), including SGRQ and COPD quality of life. Only one study reports all withdrawals [63]. Eight studies report adverse events of any cause with reason provided. One study reports withdrawals due to lack of efficacy [56]. Thirteen studies report none of the primary and secondary outcomes.

#### 3.2.7. Language

One full text is written in English. The remaining 99 full texts are written in Chinese and are translated by KH (myself).

### 3.3. Description of Excluded Studies

Two hundred and twenty-three studies are excluded after reading the full-text articles. Detailed exclusion reasons for each study are shown in Appendix 7.

Sixty-four studies (29%) are excluded because the participants are not stable COPD patients, or there is no evidence to indicate the disease phase.

Ninety-seven studies (43%) are excluded due to interventions. Reasons include that (1) CHM is not administered in the form of decoction or granules, (2) intervention group does not combine with conventional treatment, (3) intervention is not CHM or acupuncture, and (4) there is more than one CHM treatment.

Nine studies (4%) are excluded in the absence of control group or conventional treatment. Twelve studies (6%) do not report the wanted primary and secondary outcomes or do not use “intention-to-treat” analysis.

Forty-one studies (18%) are not included in the light of study designs, with reasons being not randomized or no such evidence.

### 3.4. Risk of Bias Summary of Included Studies

The risk of bias summary diagram shows the risk of bias for all included studies from *low* to *unclear* (Table 4). No study shows a low risk of bias in all six domains. Two studies (2%) show a high risk of bias in one domain [51, 56]. Ninety-nine studies (99%) display unclear risk of bias in four domains and one study (1%) shows unclear risk in two domains [63].

#### 3.4.1. Random Sequence Generation (Selection Bias)

All studies describe their randomization methods which are mostly random number generation by 1 : 1 ratio (low risk).

#### 3.4.2. Allocation Concealment (Selection Bias)

All studies do not report information about the concealment method of what types of treatment are given to participants (unclear risk).

#### 3.4.3. Blinding of Participants and Personnel (Performance Bias)

Ninety-nine studies (99%) provide no information on how participants and research personnel are blinded (unclear risk). One study reports adequately the blinding procedures of both participants and personnel (low risk) [63].

#### 3.4.4. Blinding of Outcome Assessors (Detection Bias)

Ninety-nine studies (99%) provide no information how participants and research personnel are blinded (unclear risk). One study reports adequately the blinding procedures of both participants and personnel (low risk) [63].

#### 3.4.5. Incomplete Outcome Data (Attrition Bias)

Ninety-nine studies (99%) do not report dropouts and withdrawals (unclear risk). One study (1%) reports withdrawal numbers but no reasons provided (unclear risk) [63].

#### 3.4.6. Selective Reporting (Reporting Bias)

Ninety-eight (98%) studies report all outcomes as prespecified in their protocol and two studies (2%) miss some outcome data (high risk) [51, 56].

### 3.5. Outcome Measures

One hundred studies are included with a total of 8,318. Forty studies report change in FEV_1_. Thirteen studies report exacerbation rate at the study endpoint. Twenty studies report COPD assessment test. Twenty-five studies report 6-minute walk test. Thirty-five studies report TCM syndrome score and sixty-two studies report TCM effective rate as dichotomous outcome. Seventeen studies report various QoL scales. Eleven studies report all withdrawals. Eight studies report adverse events of any cause with reason provided. One study reports withdrawals due to lack of efficacy. Thirteen studies report none of the primary and secondary outcomes.

#### 3.5.1. Primary Outcome: Change in FEV_1_

**Figure 2:**
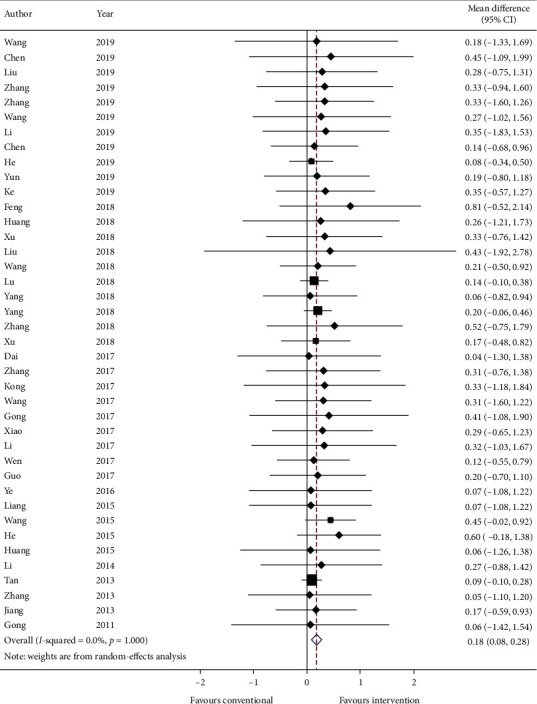
Forest plot of change in FEV_1_. CI: confidence intervals.

Forty studies report the mean and standard error (SE) of FEV_1_ (in liters) at baseline and endpoint of the study. Data are converted to standard deviation (SD) and meta-analyzed in Figure 2. The results are presented as mean change in FEV_1_ and SD. The effect estimate is 0.18 (95% CI: 0.08, 0.28; *p* ≤ 0.001) with *I*^2^ = 0.0% (*p*=1.000), which means intervention significantly increases FEV_1_ by 0.18 liter with zero heterogeneity.

GRADE: the overall quality of this evidence is judged to be moderate, downgraded once for risk of bias and once for imprecision.

#### 3.5.2. Primary Outcome: Exacerbation Rate

**Figure 3:**
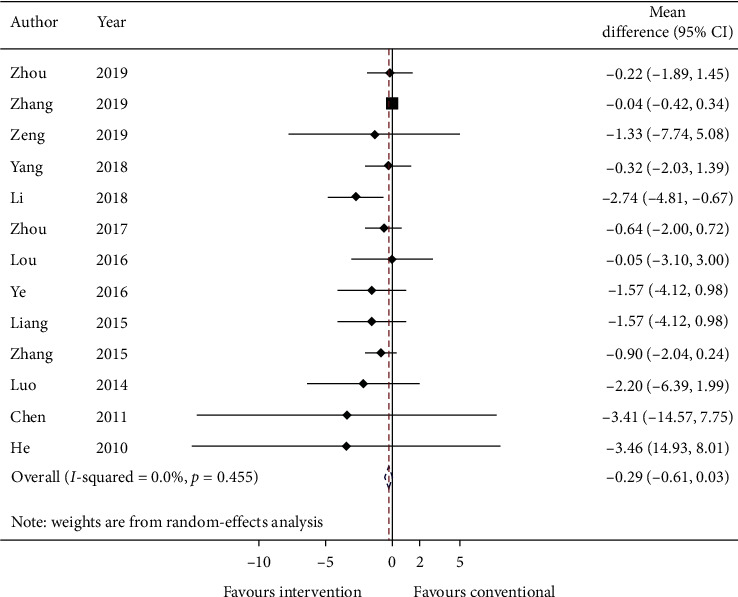
Forest plot of exacerbation rate. CI: confidence intervals.

Thirteen studies reported the mean exacerbation rate and standard error at the endpoint of the study. Data are converted to standard deviation (SD) and meta-analyzed in Figure 3. The effect estimate was −0.29 (95% CI: −0.61, 0.03; *p*=0.075733) with *I*^2^ = 0.0% (*p*=0.455).

GRADE: the overall quality of this evidence is judged to be very low, downgraded once for risk of bias, once for imprecision, and once for too few data from included studies.

#### 3.5.3. Primary Outcome: COPD Assessment Test

**Figure 4:**
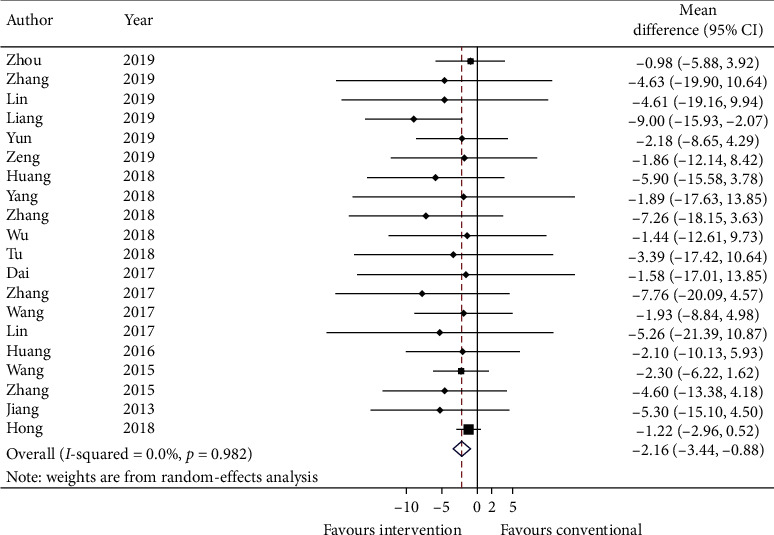
Forest plot of COPD assessment test. CI: confidence intervals.

Twenty studies report COPD assessment test (CAT) with mean and standard error. Data are converted to standard deviation (SD) and meta-analyzed in Figure 4. The effect estimate is −2.16 (95% CI: −3.44, −0.88; *p* ≤ 0.001) with *I*^2^ = 0.0% (*p*=0.982). Adding on CHM with conventional medicine significantly reduces the score of CAT.

GRADE: the overall quality of this evidence is judged to be low, downgraded once for risk of bias and once for imprecision.

#### 3.5.4. Primary Outcome: Adverse Events of Any Cause

Eight studies report adverse events of any cause and data are summarized in Table 5. Of these, four studies have no adverse events throughout the study period [28, 66–68]. Wang reports 2 cases of nausea, Liu reports 1 case of mouth dryness, and Huang reports 3 cases of acute exacerbation with hospitalization. Hong reports 2 epigastric discomfort and 1 diarrhea in the control group and 1 pale yellow phlegm in the intervention group.

GRADE: the overall quality of this evidence is judged to be low, downgraded once for risk of bias and once for imprecision.

#### 3.5.5. Secondary Outcome: Quality of Life

Seventeen studies reported quality of life using different scales. Of these, nine studies used St. George's Respiratory Questionnaire (SGRQ) [69–77]. Eight studies used COPD-Quality of Life (COPD-QoL) questionnaire [54, 67, 78–83].

**Figure 5:**
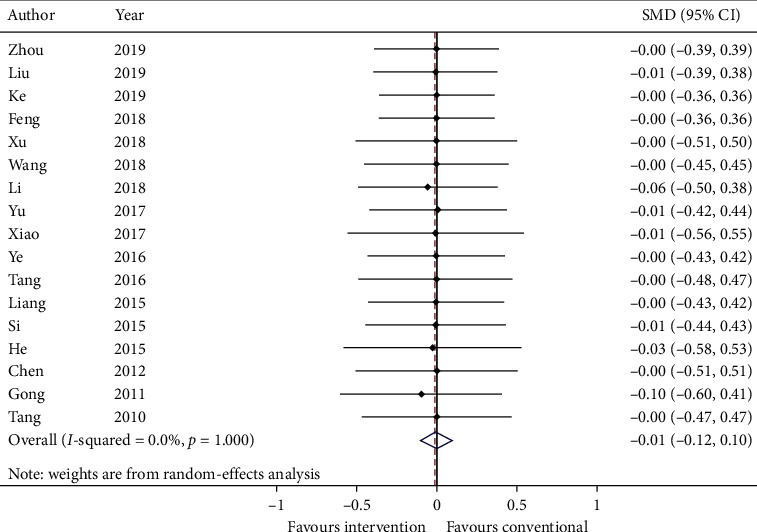
Forest plot of quality of life. CI: confidence intervals; SMD: standardized mean difference.

Standardized mean difference (SMD) and standard deviation (SD) are calculated and meta-analyzed in Figure 5. The summary estimate was −0.01 (95% CI: −0.12, 0.10; *p*=0.858723) with heterogeneity = 0.0% (*p*=1.000).

GRADE: the overall quality of this evidence is judged to be very low, downgraded once for risk of bias, once for inconsistency, and once for imprecision.

#### 3.5.6. Secondary Outcome: TCM Syndrome Score and Effective Rate

**Figure 6:**
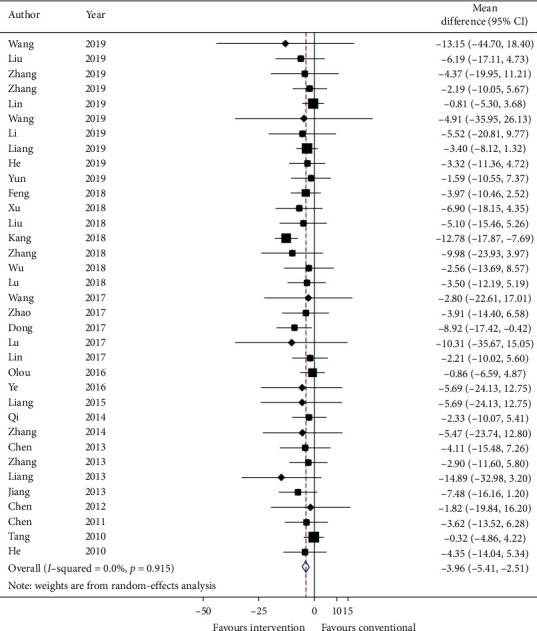
Forest plot of TCM syndrome score. CI: confidence intervals.

**Figure 7:**
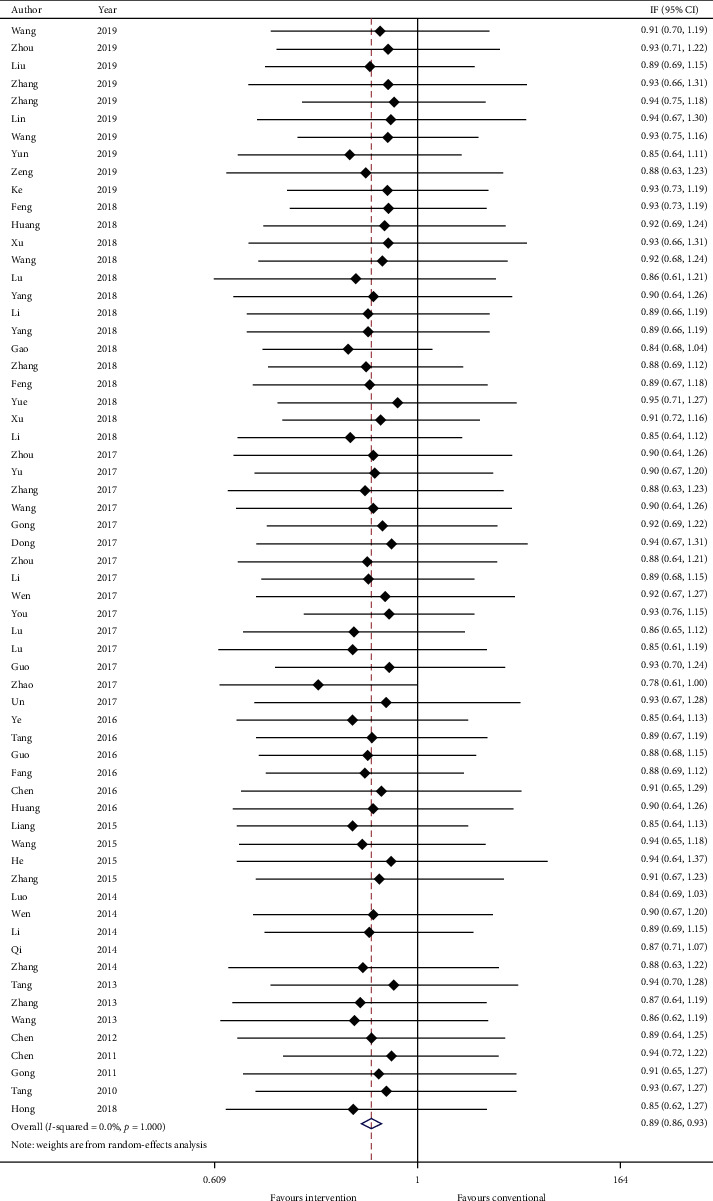
Forest plot of TCM effective rate. CI: confidence intervals.

Thirty-five studies reported total TCM syndrome score in mean plus standard error and sixty-two studies reported TCM effective rate as a number of events in each group. Data were converted to mean plus standard error and risk ratio. Meta-analysis results are presented in Figures 6 and 7, respectively. The total TCM syndrome score was reduced after adding CHM (effect estimate: MD: −3.96, 95% CI: −5.41, −2.51, *p* < 0.00001) with *I*^2^ = 0.0% (*p*=0.915). The effect estimate for TCM effective rate was 0.89 (95% CI: 0.86, 0.93, *p* < 0.00001) with heterogeneity = 0.0% (*p*=1.000).

GRADE: the overall quality of this evidence is judged to be moderate, downgraded once for risk of bias and once for imprecision.

#### 3.5.7. Secondary Outcome: 6-Minute Walk Test

**Figure 8:**
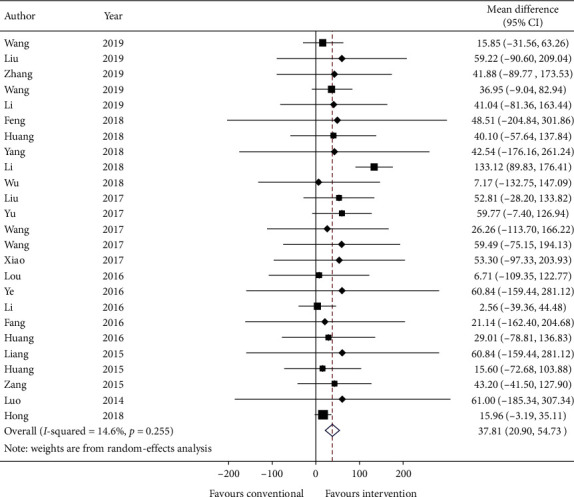
Forest plot of 6-minute walk test. CI: confidence intervals.

Twenty-five studies reported 6-minute walk test and data were reported as mean distance (in meters) and standard error. Data are transformed to standard deviation (SD) and meta-analyzed in Figure 8. The effect estimate was 37.81 (95% CI: 20.90, 54.73; *p* ≤ 0.001) favoring intervention. The heterogeneity is relatively low with *I*-squared = 14.6% (*p*=0.255).

GRADE: the overall quality of this evidence is judged to be low, downgraded once for risk of bias and once for imprecision.

### 3.6. Publication Bias

**Figure 9:**
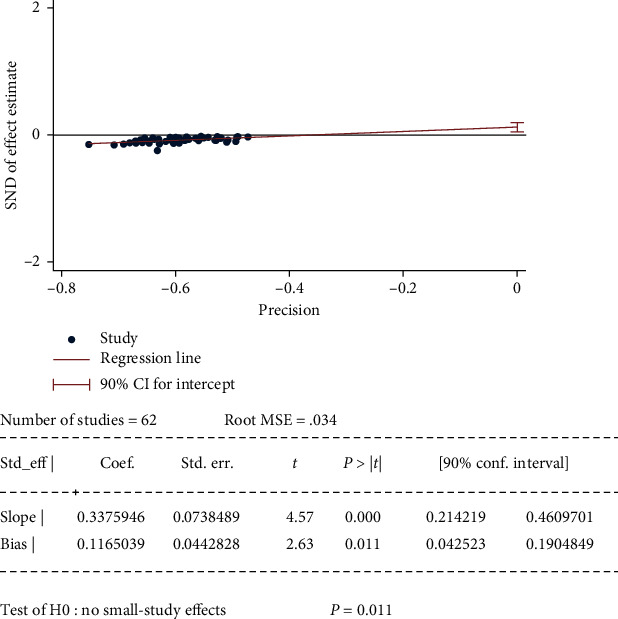
Egger's test for publication bias.

Publication bias was examined using Egger's test in TCM effective rate from 62 studies. The *p* value is 0.011 (Figure 9), which means there was a small study effect and might influence the interpretation of the summary estimate.

## 4. Discussion

### 4.1. Summary of Evidence

This systematic review evaluates the available evidence in English and Chinese of combining Traditional Chinese medicine (including Chinese herbal medicine and acupuncture) with conventional medicine on treating stable chronic obstructive pulmonary disease patients. Although there is no high-quality evidence identified, we have low-to-moderate quality randomized controlled trials with 8291 participants to suggest that it might be beneficial to incorporate TCM into conventional treatment. This review included 100 double-blinded, randomized controlled trials (8291 participants), with one comparison arm: Chinese herbal medicine plus conventional medicine versus conventional medicine only. The overall risks of bias for these studies are low to unclear. Reasons include (1) unclear reporting of allocation concealment, (2) no detailed information on blinding of participants, personnel, and outcome assessors, and (3) lack of methods reporting how to deal with missing data.

For primary outcomes, there are 40 studies showing the addition of TCM improved the force expiratory volume in 1 s (mean change: 0.18 (L), 95% CI: 0.08, 0.28, *I*^2^: 0.0%). But there are few reports about exacerbation rate, only 13 studies were meta-analyzed and the summary effect showed no reduction in acute exacerbation (mean change: −0.29, 95% CI: −0.61, 0.03, *I*^2^: 0.0%). There are also limited data (20 studies), suggesting TCM is beneficial by reducing COPD assessment test score (mean change: −2.16, 95% CI: −3.44, −0.88, *I*^2^: 0.0%) when compared to conventional medicine only.

Only eight studies reported a total of 10 adverse events, including gastrointestinal symptoms such as nausea, mouth dryness, epigastric discomfort, diarrhea, and respiratory symptoms such as pale yellow phlegm and acute exacerbation with hospitalization. Ten studies were excluded from this review because they did not perform “intention-to-treat” analysis when facing withdrawals. Only one study reported 2 withdrawals in the TCM + CT group and 3 in the CT group, which were mostly lost during treatment. No studies reported adverse effects of either Chinese herbal medicine or acupuncture.

For secondary outcomes, there is limited evidence to show that TCM can improve patients' quality of life, with only 17 studies and different scales used. So we calculated the standardized mean difference and the effect estimate was −0.01 (95% CI: −0.12, 0.10, *I*^2^: 0.0%), which did not show any improvement. There are thirty-five studies reporting the change in TCM syndrome score. The symptoms and signs were less severe in the CHM + CT group (mean change: −3.96, 95%CI: -5.41, −2.51, *I*^2^ = 0.0%). Merely sufficient evidence (62 studies) showed that TCM was more effective combined with conventional treatment (RR: 0.89, 95% CI: 0.86, 0.93, *I*^2^ = 0.0%). The distance walked in 6 minutes was increased by 37.81 meters (95% CI: 20.90, 54.73, *I*^2^: 14.6%) in the intervention group when compared to the control group with 25 studies.

GRADE: the outcomes change in FEV1, TCM syndrome score, and effective rate were rated as moderate. This implies there is some confidence in the effect estimate, and the true effect is likely to be close to the estimate of effect, but there is a chance that it is substantially different. Future research is likely to have an important impact on the confidence in the estimate of effect and may change the estimate [40]. The outcomes COPD assessment test, adverse events of any cause, all withdrawals of study participants, and 6-minute walk test were judged to be low-quality evidence. This implies there is limited confidence in the effect estimate and the true effect may be substantially different from the estimate of effect. Future research is very likely to change the estimate of effect or impact the confidence in the estimate of effect [40]. The outcomes exacerbation rate, withdrawals due to a lack of efficacy, and quality of life were rated as very low-quality evidence. This implies there is very little confidence in the effect estimate between types of treatments and the true effect is likely to be substantially different from the estimate of effect. The results of these outcomes are very uncertain and the effect estimate is not accurate enough to recommend any use of Chinese herbal medicine [40]. In summary, the body of evidence suggests that adding Chinese herbal medicine to conventional treatment may be beneficial in stable COPD patients.

### 4.2. Strengths of This Review

A major strength of this review is we strictly included level 1 evidence of double-blinded RCTs only. This review does not include studies where participants have both acute exacerbated and stable chronic obstructive pulmonary disease and any complicated diseases. Previous systematic reviews report either one single herb extract or formulae, or one specific TCM syndrome-related treatment method [32, 67, 84–91]. However, this is not the usual practice of traditional Chinese medicine. In both outpatient and inpatient settings, TCM uses various formula or acupoints combination according to syndrome differentiation even in the same stable COPD population. This review identifies the add-on effect of any formation of Chinese herbal medicine with different TCM syndromes in comparison with conventional medicine only. This fills the gaps between the normal use of TCM and contemporary research in treating stable COPD patients.

This review identifies the need for high-quality double-blinded randomized controlled trials with more participants in each arm and more detailed reporting of research methods (randomization and blinding of participants and accessors).

### 4.3. Limitations of This Review

The key methodological limitation to this review was the language restriction, and only Chinese and English literature studies were searched. We believed there were studies written in other languages such as Korean and Japanese, where Chinese herbal medicine and acupuncture were often used. A more comprehensive review might be needed to include all languages.

A second limitation to this review was the broad inclusion criteria of herbal formulae or drugs and treatment duration. This would limit the specificity analysis of a certain herb or formulae, as we generally analyzed CHM as an adjunct treatment to conventional medicine. This might also limit the usage of CHM in clinical situation because we would need a registered Chinese medicine practitioner to diagnose with syndrome differentiation. Subgroup analyses were planned but could not be done in this review as there were too many combinations of herbal drugs and treatment durations. It was unable to investigate whether any of these variables affected treatment efficacy.

A third limitation of this review was lacking studies with sample sizes larger than or equal to 200 participants per arm.

## 5. Conclusions

### 5.1. Implications for Clinical Practice

Traditional Chinese medicine has been used to treat COPD-related symptoms over decades, yet its effectiveness and safety remain uncertain. Previous clinical studies reported either one single Chinese herbal formulae or one specific Chinese medicine treatment method. Considering there are numerous formulae or combinations of herbs that might be beneficial to stable COPD patients, it is difficult to use current evidence to guide the use of TCM in addition to conventional medicine.

Although we saw some statistical significance in several outcome parameters, it did not mean that there are real treatment effects clinically. Our data suggested that TCM combined with conventional treatment was beneficial in FEV_1_, COPD assessment test, 6-minute walk distance, and TCM syndrome statistically. Clinicians may consider incorporating TCM into the mainstream medical system with reference to their own clinical experience.

### 5.2. Implications for Research

High-quality randomized controlled clinical trials or pragmatic trials are needed. In order to provide real information for Chinese medicine practitioners, TCM theories and diagnoses must be taken into account when designing clinical research protocols and conducting trials.

More advanced analyses, like individual participant data and network meta-analysis, can be applied to provide more information on different combinations of herbal drugs and/or acupuncture and generate personalized evidence with reference to TCM theories.

## Figures and Tables

**Table 1:** Search terms used in PubMed.

Number	Search terms
1	Randomized controlled trial
2	RCT
3	Randomized
4	Randomly
5	Trial
6	Groups
7	Controlled clinical trial
8	1 or 2-7
9	Chronic obstructive pulmonary disease
10	COPD
11	Chronic obstructive airway disease
12	Chronic obstructive respiratory disease
13	Chronic bronchitis
14	Emphysema
15	Chronic airflow obstruction
16	9 or 10-15
17	Chinese Medicine
18	Chinese Herbal Medicine
19	CHM
20	Traditional Chinese Medicine
21	TCM
22	Traditional medicine
23	Herb*∗*
24	Herb*∗*medicine
25	Plant medicine
26	Herb formula
27	Herb decoction
28	17 or 18-27
29	Acupuncture
30	Acupoint*∗*
31	Needling
32	Dry needling
33	29, 30–32
34	8 and 16 and 28 and 33

**Table 2:** Basic characteristics of included studies.

Author	Year	Sample size (intervention/control)	Age (years) (course of disease (years))		Gender (male/female)		Intervention#	Control	Treatment duration (weeks)	Baseline difference	Quality control
			Control	Intervention	Control	Intervention					
Wang	2019	48/48	63.04 ± 8.39 (12.17 ± 3.51)	62.87 ± 9.35 (12.63 ± 3.84)	25/23	27/21	MSZYQD + CT	CT	12	NSD	NR
Zhu	2019	45/45	63.6 ± 8.4 (13.5 ± 7.4)	61.6 ± 8.5 (13.4 ± 7.3)	23/22	25/20	YQYYTBLMZY + CT	CT	4	NSD	NR
Chen	2019	64/64	67.89 ± 9.58 (3.12 ± 0.45)	66.99 ± 10.77 (3.21 ± 0.36)	31/33	32/32	MXBFD + CT	CT	12	NSD	NR
Zhou	2019	50/50	67.50 ± 6.51 (NR)	63.74 ± 6.5 (NR)	31/19	32/18	BSNQG + CT	CT	8	NSD	NR
Liu	2019	51/52	61.75 ± 8.2 (6.05 ± 2.33)	62.40 ± 0.31 (5.93 ± 2.47)	34/17	36/16	BFHXD + CT	CT	12	NSD	NR
Zhang	2019	30/30	64.02 ± 3.49 (8.02 ± 2.64)	63.82 ± 3.67 (7.85 ± 2.71)	16/14	17/13	MSZJQD + CT	CT	8	NSD	NR
Zhang	2019	72/72	45.18 ± 6.30 (7.25 ± 1.99)	45.30 ± 6.12 (7.38 ± 1.99)	41/31	45/27	SMZYYFD + CT	CT	12	NSD	NR
Lin	2019	33/33	68.24 ± 9.76 (22.30 ± 2.75)	70.62 ± 9.38 (23.52 ± 2.89)	20/13	21/12	YSBFG + CT	CT	24	NSD	NR
Wang	2019	73/73	62.05 ± 5.13 (6.40 ± 1.25)	63.32 ± 4.59 (6.02 ± 1.14)	47/16	44/29	SMQFWSHTD + CT	CT	12	NSD	NR
Jin	2019	30/30	NR	NR	NR	NR	SLBZDAAS + CT	CT	12	NSD	NR
Li	2019	40/40	68.25 ± 15.21 (7.82 ± 2.53)	(8.15 ± 2.41)	25/15	23/17	FZHTQYM + CT	CT	12	NSD	NR
Liang	2019	40/40	62 ± 10 (6 ± 5)	64 ± 10 (6 ± 5)	23/17	22/18	GBQFD + CT	CT	12	NSD	NR
Wang	2019	30/30	NR	NR	NR	NR	SLBZDAAS + CT	CT	12	NSD	NR
Chen	2019	38/38	67.52 ± 3.22 (3.03 ± 0.38)	67.56 ± 3.24 (3.05 ± 0.37)	19/19	20/18	SQBFD + CT	CT	8	NSD	NR
He	2019	38/38	62.5 ± 0.5	62.4 ± 0.6	21/17	20/18	SQBFD + CT	CT	8	NSD	NR
Yun	2019	42/42	63.5 ± 11.2 (12.5 ± 4.6)	67.2 ± 14.1 (10.5 ± 5.2)	19/23	22/20	SQGBD + CT	CT	24	NSD	NR
Zeng	2019	30/30	64.43 ± 6.95 (9.5 ± 2.74)	66.20 ± 7.27 (9.0 ± 2.03)	19/11	16/14	LWBQG + CT	CT	32	NSD	NR
Ke	2019	58/58	45.6 ± 7.1 (3.14 ± 0.96)	44.8 ± 6.9 (3.27 ± 0.88)	31/27	35/23	JPYFD + CT	CT	3-4	NSD	NR
Feng	2018	60/60	60.12 ± 2.76 (8.14 ± 2.23)	59.42 ± 2.56 (7.58 ± 1.24)	36/24	38/22	QTJFD + CT	CT	12	NSD	NR
Huang	2018	40/40	65.56 ± 5.83 (5–20)	64.76 ± 6.46 (6–17)	29/11	30/10	JSLJD + THSWD + CT	CT	8	NSD	NR

Xu	2018	30/30	61.4 ± 4.9 (12.3 ± 1.7)	62.6 ± 5.5 (12.6 ± 1.5)	15/15	14/16	BFHXD + CT	CT	28	NSD	NR
Liu	2018	55/55	51.97 ± 17.34 (9.2 ± 5.3)	52.61 ± 16.99 (9.4 ± 4.8)	35/20	34/21	BFHXD + CT	CT	12	NSD	NR
Kang	2018	75/75	61.25 ± 11.57	62.2 ± 12.65	44/31	52/23	BZYQD + CT	CT	24	NSD	NR
Wang	2018	38/38	65.44 ± 6.85 (6.23 ± 2.35)	65.96 ± 6.57 (6.43 ± 2.24)	19/19	20/18	YQBFD + CT	CT	4	NSD	NR
Lu	2018	29/29	58.3 ± 1.3	59.2 ± 1.2	16/13	15/14	LJZD + CT	CT	8	NSD	NR
Yang	2018	30/30	57.14 ± 10.67 (9.92 ± 6.02)	56.75 ± 11.14 (10.36 ± 5.74)	21/9	20/10	FFGSD + CT	CT	12	NSD	NR
Li	2018	40/40	61.2 ± 2.1 (5.4 ± 1.1)	63.4 ± 1.8 (5.1 ± 1.0)	28/12	29/11	MZFWDD + CT	CT	16	NSD	NR
Yang	2018	40/40	75.0 ± 4.5 (5.0 ± 2.2)	74.5 ± 5.0 (5.3 ± 2.1)	28/12	27/13	PKHTD + CT	CT	12	NSD	NR
Gao	2018	80/80	59.7 ± 6.4 (16.4 ± 2.1)	58.8 ± 5.9 (15.9 ± 2.7)	54/26	51/29	SGDHP + CT	CT	8	NSD	NR
Zhang	2018	60/60	62.39 ± 5.43 (13.95 ± 3.79)	64.36 ± 5.65 (13.19 ± 3.99)	37/23	34/26	SGBFD + CT	CT	12	NSD	NR
Wu	2018	15/15	51.46 ± 8.42 (10.01 ± 7.19)	53.25 ± 7.51 (9.85 ± 8.15)	8/7	6/9	SLBZD + CT	CT	12	NSD	NR
Lu	2018	40/40	64.49 ± 8.22 (7.82 ± 0.92)	65.28 ± 8.31 (7.79 ± 0.90)	25/15	26/14	SQBFD + CT	CT	4	NSD	NR
Feng	2018	42/42	63.1 ± 8.7 (NR)	64.7 ± 9.2 (NR)	24/18	25/17	SQBFD + CT	CT	12	NSD	NR
Yue	2018	43/42	528.±4.9 (12.3 ± 3.7)	53.6 ± 3.2 (11.4 ± 2.2)	25/18	23/19	SQBFD + CT	CT	12	NSD	NR
Xu	2018	60/60	65.65 ± 1.36 (10 ± 0.27)	66.35 ± 2.16 (10 ± 0.65)	38/22	36/24	MBXXXD + CT	CT	8	NSD	NR
Tu	2018	30/30	60.00 ± 6.80 (11.53 ± 4.90)	62.60 ± 5.74 (11.92 ± 5.02)	29/1	28/2	MLJZD + CT	CT	12	NSD	NR
Li	2018	44/44	69.58 ± 1.02 (12.1 ± 3.4)	69.42 ± 1.03 (13.2 ± 4.5)	26/18	28/16	YFBJD + CT	CT	4	NSD	NR
Dai	2017	36/36	64 ± 7.2 (18.64 ± 10.61)	63.33 ± 6.11 (15.61 ± 8.05)	19/17	18/18	SSSQD + CT	CT	12	NSD	NR
Zhou	2017	30/30	72.05 ± 7.62(6.03 ± 1.09)	71.89 ± 7.56(6.57 ± 1.14)	17/12	18/12	MZFWDF + CT	CT	12	NSD	NR
Liu	2017	40/40	64.21 ± 6.91(13.56 ± 8.62)	66.17 ± 6.02(12.89 ± 7.94)	27/13	25/15	ZYZYF + CT	CT	4.29	NSD	NR
Yu	2017	42/41	63.34 ± 5.19 (NR)	63.34 ± 5.19 (NR)	20/21	21/21	BZYQD + CT	CT	24	NSD	NR
Zhang	2017	50/50	52.02 ± 3.10(12.80 ± 2.11)	53.23 ± 3.02(13.30 ± 2.01)	27/23	28/22	SZYQD + CT	CT	2	NSD	NR

Kong	2017	30/30	56.5 ± 13.5(9.5 ± 5.5)	55 ± 13(9 ± 6)	19/11	17/13	BFFCD + CT	CT	12	NSD	NR
Wang	2017	30/30	69.43 ± 4.897(5.80 ± 2.709)	68.00 ± 6.119(6.10 ± 2.746)	18/12	21/9	ILANKM + CT	CT	8.57	NSD	NR
Wang	2017	42/41	66.43 ± 7.76(5.73 ± 1.64)	67.08 ± 7.43(6.02 ± 1.58)	24/17	26/17	BFHXD + CT	CT	24	NSD	NR
Zhao	2017	42/42	61.32 ± 10.03 (NR)	62.99 ± 9.10 (NR)	24/18	23/19	BFD + CT	CT	8	NSD	NR
Gong	2017	45/44	59.62 ± 5.97(19.61 ± 4.85)	59.63 ± 5.96(19.62 ± 5.97)	24/20	24/21	BFJPYSD + CT	CT	4	NSD	NR
Dong	2017	33/32	66.3 ± 8.3 (NR)	66.5 ± 8.1 (NR)	15/17	16/17	GJDCP + CT	CT	12	NSD	NR
Zhou	2017	35/35	53.7 ± 10.9 (NR)	53.7 ± 10.9 (NR)	34/36	34/36	SMYFPCD + CT	CT	12	NSD	NR
Xiao	2017	25/25	64.3 ± 5.7(8.5 ± 2.3)	64.5 ± 5.3(8.3 ± 2.1)	18/7	16/9	SMWBPSD + CT	CT	12	NSD	NR
Li	2017	50/50	70.06 ± 3.09(6.12 ± 1.12)	70.61 ± 3.11(6.02 ± 1.05)	26/24	27/23	SMYYQFD + CT	CT	1	NSD	NR
Wen	2017	33/34	56.85 ± 5.47(8.36 ± 3.63)	56.85 ± 5.47(8.36 ± 3.63)	NR	NR	FLK + CT	CT	12	NSD	NR
You	2017	83/83	63.75 ± 5.91(7.08 ± 1.51)	63.37 ± 5.87(6.88 ± 1.53)	46/37	50/33	YQHTQYTLD + CT	CT	8.57	NSD	NR
Lu	2017	38/38	64.20 ± 13.40 (NR)	64.20 ± 13.40 (NR)	22/16	19/19	WBFSM + CT	CT	24	NSD	NR
Yang	2017	40/40	NR	NR	34/6	31/9	FFGSD + CT	CT	12	NSD	NR
Lu	2017	44/44	NR	NR	NR	NR	PCGBD + CT	CT	24	NSD	NR
Lu	2017	31/31	63.5 ± 4.1(4.2 ± 0.8)	64.2 ± 4.2(4.0 ± 0.6)	17/14	18/13	SLBZPAAS + CT	CT	12	NSD	NR
Li	2017	38/38	40.32 ± 3.12 (NR)	40.65 ± 3.08 (NR)	21/17	22/16	MSGP + CT	CT	8	NSD	NR
Guo	2017	46/46	59.1 ± 5.9 (NR)	58.±5.7 (NR)	27/19	29/17	YYQFD + CT	CT	4	NSD	NR
Zhao	2017	50/50	67.45 ± 3.14(4.8 ± 1.1)	65.32 ± 2.25(4.7 ± 1.2)	34/16	38/12	SMLRD + CT	CT	2	NSD	NR
Lin	2017	34/34	60.49 ± 8.26(7.01 ± 2.39)	60.49 ± 8.26(6.89 ± 2.60)	19/15	19/15	MLJZD + CT	CT	12	NSD	NR
Lou	2016	60/59	71.2 ± 5.7(19.23 ± 4.53)	72.35 ± 4.77(18.42 ± 3.37)	36/23	35/35	JQNSM + CT	CT	12	NSD	NR
Ye	2016	42/42	63.14 ± 12.08(6.84 ± 2.99)	62.47 ± 11.08(7.01 ± 3.68)	24/18	22/20	TBFSM + CT	CT	12	NSD	NR
Bian	2016	25/25	60.3 ± 5.7(16.4 ± 3.6)	60.3 ± 5.7(16.4 ± 3.6)	NR	NR	BSFCD + CT	CT	12	NSD	NR

Li	2016	30/30	61.33 ± 11.50(14.12 ± 5.3)	62.2 ± 12.67(12.5 ± 4.88)	16/14	19,11	ZLFSQXDGFTJ + CT	CT	8	NSD	NR
Tang	2016	34/34	65.87 ± 9.08 (NR)	63.77 ± 8.64 (NR)	20/14	23/11	ZCG + CT	CT	8	NSD	NR
Guo	2016	49/49	69.3 ± 2.5(5.4 ± 0.7)	70.2 ± 2.3(5.6 ± 0.5)	28/21	27/22	MZFWDF + CT	CT	12	NSD	NR
Fang	2016	59/59	64.02 ± 11.15(6.79 ± 2.14)	63.87 ± 10.62(6.32 ± 2.02)	36/23	35/24	SLBZPAAS + CT	CT	12	NSD	NR
Chen	2016	30/30	66.1 ± 5.8(6.3 ± 1.6)	65.6 ± 5.1(6.5 ± 1.8)	17/13	19/11	LJF + CT	CT	8	NSD	NR
Hu	2016	30/30	67.90 ± 7.34(16.47 ± 6.95)	68.63 ± 6.49(14.87 ± 9.07)	19/11	18/12	BFDAAS + CT	CT	8	NSD	NR
Liang	2015	42/42	63.14 ± 12.08(6.84 ± 2.99)	62.47 ± 11.08(7.01 ± 3.68)	24/18	22/20	TBFSM + CT	CT	12	NSD	NR
Wang	2015	37/37	67.13 ± 6.95(3.92 ± 0.39)	66.72 ± 7.92(3.85 ± 0.44)	15/22	18/19	BFYSD + CT	CT	1	NSD	NR
Si	2015	40/40	70.43 ± 8.73(16.30 ± 5.19)	72.04 ± 9.36(18.30 ± 5.84)	22/18	25/15	BFJPYSD + CT	CT	NR	NSD	NR
Wang	2015	36/36	60.51 ± 11.03(14.52 ± 5.96)	60.81 ± 8.18(14.56 ± 6.32)	22/14	21/15	BZGWHJ + CT	CT	8	NSD	NR
Li	2015	38/38	57.18 ± 9.92 (NR)	56.63 ± 10.23 (NR)	23/15	20/18	YQBSHXF + CT	CT	12	NSD	NR
He	2015	25/25	NR	NR	NR	NR	SLBZG + CT	CT	36	NSD	NR
Huang	2015	40/41	67.28 ± 4.30(8.9 ± 7.8)	65.36 ± 2.10(8.8 ± 7.1)	25/16	23/17	KFZY + CT	CT	36	NSD	NR
Zhang	2015	45/30	63.2 ± 11.5 (NR)	63.8 ± 10.7 (NR)	18/22	29/16	SXDAAS + CT	CT	12	NSD	NR
Luo	2014	80/80	66.8 (15.49)	64.8 (16.32)	62/18	64/16	BFPCD + CT	CT	12	NSD	NR
Wen	2014	40/40	68.3 ± 9.0(15.4 ± 3.6)	68.6 ± 9.2(15.7 ± 3.8)	28/12	30/10	BFDAAS + CT	CT	8	NSD	NR
Li	2014	49/49	61.37 ± 4.18(10.6 ± 8.9)	63.52 ± 3.67(11.4 ± 7.3)	30/19	32/17	BFNSF + CT	CT	3	NSD	NR
Qi	2014	80/80	55.9 ± 3.9(8.2 ± 3.1)	56.3 ± 4.2(7.9 ± 4.2)	46/34	44/36	WSBFHTT + CT	CT	8	NSD	NR
Zeng	2014	43/42	66.9 ± 7.9(14.8 ± 3.9)	67.4 ± 7.2(15.3 ± 4.5)	29/13	28/15	MSZYQD + GZLMD + CT	CT	12	NSD	NR
Zhang	2014	30/30	40.46 ± 16. (NR)	45.35 ± 15.32 (NR)	12/18	7/23	QZYQDG + CT	CT	2	NSD	NR
Chen	2013	30/30	63.51 ± 11.24(10.86 ± 7.54)	62.54 ± 12.56(11.61 ± 8.44)	18/12	16/14	BFYSQTD + CT	CT	42	NSD	NR
Tan	2013	45/34	NR	NR	NR	NR	BZYQM + CT	CT	2.14	NSD	NR

Zhang	2013	33/33	63.57 ± 11.47(33.57 ± 10.97)	62.03 ± 10.94(33.26 ± 9.41)	19/14	20/13	QYJDF + CT	CT	4	NSD	NR
Liang	2013	40/40	NR	NR	38/2	33/7	RFJPBSD + CT	CT	12	NSD	NR
Jiang	2013	30/30	61.7 ± 3.2 (NR)	64.0 ± 2.1 (NR)	19/11	17/13	SLBZG + CT	CT	24	NSD	NR
Wang	2013	30/30	NR	NR	NR	NR	SLBZDAAS + CT	CT	12	NSD	NR
Dai	2013	40/40	NR	NR	NR	NR	SMBPYQD + CT	CT	12	NSD	NR
Chen	2012	30/30	60.81 ± 8.57 (NR)	65.63 ± 7.43 (NR)	21/9	23/7	GBQTHYD + CT	CT	24	NSD	NR
Chen	2011	50/50	67.73 ± 6.11(9.75 ± 3.56)	69.51 ± 5.41(9.45 ± 4.12)	41/9	38/12	FGFZM + CT	CT	12	NSD	NR
Gong	2011	30/30	68.4 ± 6.2 (NR)	67.4 ± 6.8 (NR)	19/11	17/13	SETGM + CT	CT	24	NSD	NR
Tang	2010	34/35	71.00 ± 10.53(15.46 ± 10.89)	72.18 ± 10.78(14.53 ± 9.15)	26/9	23/11	JPYSD + CT	CT	12	NSD	NR
He	2010	49/49	NR	NR	NR	NR	SETGM + CT	CT	24	NSD	NR
Wang	2009	36/28	61.5 ± 4.8(15.6 ± 4.7)	62.1 ± 5.3(16.3 ± 5.1)	21/7	25/11	SMBFTFD + CT	CT	24	NSD	NR
Wang	2008	32/26	61.5 ± 4.8(15.6 ± 4.7)	62.1 ± 5.3(16.3 ± 5.1)	20/6	23/9	BFTFD + CT	CT	NR	NSD	NR
Jiang	2008	30/25	63.2 ± 5.3(13.12 ± 3.38)	61.6 ± 6.1(15.25 ± 4.01)	17/8	23/7	SMYQHXD + CT	CT	24	NSD	NR
Hong	2018	30/30	68.59 ± 7.72 (15.70 ± 5.59)	67.93 ± 7.78 (14.57 ± 5.50)	27/1	28/0	YFN + CT	CT	8	NSD	YES

NSD = not significantly different; NR = not reported; CT = conventional treatment. # Detailed description of CHM in Appendix 6.

**Table 3:** Availability of outcome indexes.

Author	Year	Available outcome index
Wang	2019	TCM syndrome score, TCM effective rate, FEV_1_, 6MWT
Zhu	2019	Nil
Chen	2019	FEV_1_
Zhou	2019	TCM effective rate, CAT, QoL, exacerbation rate
Liu	2019	TCM syndrome score, TCM effective rate, FEV_1_, 6MWT, QoL, adverse events
Zhang	2019	TCM syndrome score, FEV_1_, CAT, TCM effective rate
Zhang	2019	TCM syndrome score, FEV_1_, 6MWT, exacerbation rate, TCM effective rate
Lin	2019	TCM syndrome score, CAT, TCM effective rate
Wang	2019	TCM syndrome score, FEV_1_, 6MWT, TCM effective rate, adverse events
Jin	2019	Nil
Li	2019	TCM syndrome score, FEV_1_, 6MWT
Liang	2019	TCM syndrome score, CAT
Wang	2019	Nil
Chen	2019	FEV_1_
He	2019	TCM syndrome score, FEV_1_
Yun	2019	TCM syndrome score, TCM effective rate, FEV_1_, CAT
Zeng	2019	TCM effective rate, exacerbation rate, CAT
Ke	2019	FEV_1,_ QoL
Feng	2018	TCM syndrome score, TCM effective rate, FEV_1_, 6MWT, QoL
Huang	2018	TCM effective rate, FEV1, CAT, 6MWT
Xu	2018	TCM syndrome score, TCM effective rate, FEV_1_, QoL
Liu	2018	TCM syndrome score, FEV_1_
Kang	2018	TCM syndrome score
Wang	2018	TCM effective rate, QoL, FEV_1_
Lu	2018	TCM effective rate, FEV_1_
Yang	2018	TCM effective rate, FEV_1_, CAT, 6MWT, exacerbation rate
Li	2018	TCM effective rate, exacerbation rate, 6MWT, QoL
Yang	2018	TCM effective rate, FEV_1_
Gao	2018	TCM effective rate
Zhang	2018	TCM effective rate, TCM syndrome score, CAT, FEV_1_
Wu	2018	TCM syndrome score, 6MWT, CAT
Lu	2018	TCM syndrome score
Feng	2018	TCM effective rate
Yue	2018	TCM effective rate
Xu	2018	TCM effective rate, FEV_1_
Tu	2018	CAT
Li	2018	TCM effective rate
Dai	2017	FEV_1_, CAT
Zhou	2017	TCM effective rate, exacerbation rate
Liu	2017	6MWT, adverse events
Yu	2017	TCM effective rate, QoL, 6MWT
Zhang	2017	FEV_1_, CAT, TCM effective rate
Kong	2017	FEV_1_
Wang	2017	TCM syndrome score, TCM effective rate, 6MWT, CAT
Wang	2017	FEV_1_, 6MWT
Zhao	2017	TCM syndrome score
Gong	2017	FEV_1_, TCM effective rate
Dong	2017	TCM syndrome score, TCM effective rate
Zjou	2017	TCM effective rate
Xiao	2017	FEV_1_, QoL, 6MWT
Li	2017	TCM effective rate, FEV_1_, adverse events
Wen	2017	TCM effective rate, FEV_1_
You	2017	TCM effective rate, adverse events
Lu	2017	Nil
Yang	2017	Nil
Lu	2017	TCM syndrome score, TCM effective rate
Lu	2017	TCM effective rate
Li	2017	Nil
Guo	2017	FEV_1_, TCM effective rate
Zhao	2017	TCM effective rate
Lin	2017	TCM effective rate, TCM syndrome score, CAT
Lou	2016	TCM syndrome score, 6MWT, exacerbation rate
Ye	2016	TCM syndrome score, QoL, TCM effective rate, FEV_1_, 6MWT, exacerbation rate
Bian	2016	Nil
Li	2016	6MWT
Tang	2016	QoL, TCM effective rate
Guo	2016	TCM effective rate
Fang	2016	TCM effective rate, 6MWT
Chen	2016	TCM effective rate
Hu	2016	TCM effective rate, 6MWT, CAT
Liang	2015	TCM syndrome score, TCM effective rate, 6MWT, QoL, FEV_1_, exacerbation rate
Wang	2015	TCM effective rate, FEV_1_
Si	2015	QoL
Wang	2015	CAT
Li	2015	Nil
He	2015	TCM effective rate, FEV_1,_ QoL
Huang	2015	FEV_1_, 6MWT, adverse events
Zhang	2015	CAT, 6MWT, TCM effective rate, exacerbation rate, adverse events
Luo	2014	6MWT, exacerbation rate, TCM effective rate, adverse events
Wen	2014	TCM effective rate
Li	2014	FEV_1_, TCM effective rate
Qi	2014	TCM effective rate, TCM syndrome score
Zeng	2014	Nil
Zhang	2014	TCM effective rate, TCM syndrome score
Chen	2013	TCM syndrome score
Tan	2013	FEV_1_, TCM effective rate
Zhang	2013	TCM effective rate, TCM syndrome score, FEV_1_
Liang	2013	TCM syndrome score, 6MWT
Jiang	2013	CAT, TCM syndrome score, FEV_1_
Wang	2013	TCM effective rate
Dai	2013	Nil
Chen	2012	TCM effective rate, TCM syndrome score
Chen	2011	TCM effective rate, FEV_1_, TCM syndrome score, exacerbation rate
Gong	2011	FEV_1_, TCM effective rate, QoL
Tang	2010	TCM syndrome score, TCM effective rate, QoL
He	2010	TCM syndrome score, exacerbation rate
Wang	2009	Nil
Wang	2008	Nil
Jiang	2008	Nil
Hong	2018	TCM effective rate, CAT, 6MWT, adverse events, all withdrawals

FEV1 = forced expiratory volume in 1 s; CAT = COPD assessment test; 6MWT = 6-minute walk test; QoL = quality of life.

**Table 4:** Risk of bias assessments of included studies.

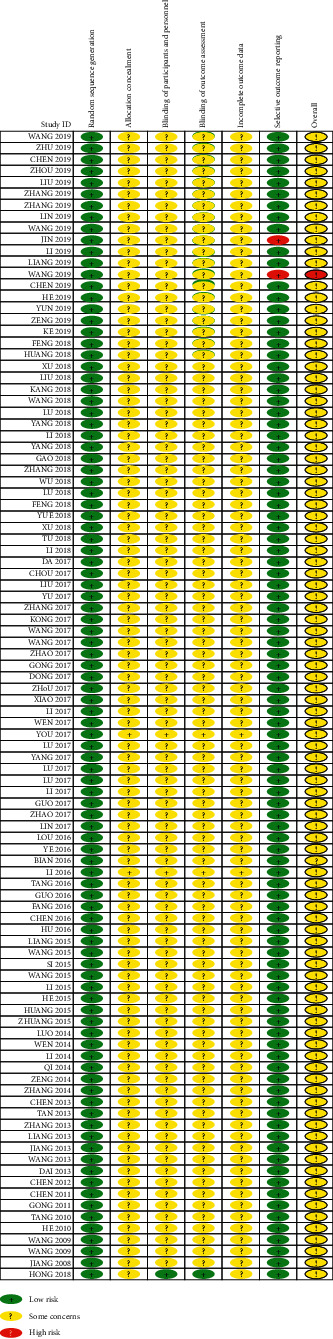

**Table 5:** Adverse events of any cause.

Study	Group (number of events)	Cause
[56]	Control (0)	—
	Intervention (2)	Nausea

[64]	Control (0)	—
	Intervention (1)	Mouth dryness

[65]	Control (2)	Acute exacerbation
	Intervention (1)	Acute exacerbation

[63]	Control (3)	2 epigastric discomfort, 1 diarrhea
	Intervention (1)	1 pale yellow phlegm

## Data Availability

The clinical data used to support the findings of this study are included within the supplementary information files.

## Abbreviations

BOLD: Burden of Obstructive Lung Diseases
CAT^TM^: COPD assessment test
CBM: Chinese Biomedical Database
CENTRAL: Cochrane Central Register of Controlled Trials
CHM: Chinese herbal medicine
CI: Confidence interval
CNKI: Chinese National Knowledge Infrastructure
COPD: Chronic obstructive pulmonary disease
CT: Conventional treatment
FEV_1_: Forced expiratory volume in first second
FVC: Forced vital capacity
GRADE: Grading of Recommendations, Assessment, Development, and Evaluation system
GOLD: Global Initiative for Chronic Obstructive Lung Disease
ICTRP: The World Health Organization International Clinical Trials Registry Platform
ITT: Intention-to-treat
LABA: Long-acting beta_2_-agonist
LAMA: Long-acting anticholinergic
MD: Mean difference
MeSH: Medical subject headings
mMRC: Modified British Medical Research Council
mRCT: metaRegister of controlled trials
PRISMA-P: Preferred Reporting Items for Systematic Review and Meta-Analysis Protocols
PROSPERO: Prospective register of systematic review
QoL: Quality of life
RCT: Randomized controlled trial
SABA: Short-acting beta_2_-agonist
SABD: Short-acting bronchodilator
SAMA: Short-acting anticholinergic
SD: Standard deviation
SE: Standard error
SGRQ: St. George's Respiratory Questionnaire
SMD: Standardized mean difference
TCM: Traditional Chinese medicine
VIP: Chinese Scientific and Technological Periodical Database
WHO: World Health Organization.

## Supplementary Materials

Supplementary Materials Appendix 1: research protocol. Appendix 2: PRISMA statement reporting standards checklist. Appendix 3: data extraction form. Appendix 4: Cochrane risk of bias tool for randomized controlled trials. Appendix 5: GRADE guidelines. Appendix 6: description of Chinese medicine. Appendix 7: characteristics of excluded studies table.
