# Supplementary material for: The Effectiveness of Traditional Chinese Medicine (TCM) as an Adjunct Treatment on Stable COPD Patients: A Systematic Review and Meta-Analysis
Source: Evid Based Complement Alternat Med. 2021 Jun 4;2021:5550332. doi: 10.1155/2021/5550332 (PMC8195656; doi:10.1155/2021/5550332)
Supplement: Supplementary Materials — Appendix 1: research protocol. Appendix 2: PRISMA statement reporting standards checklist. Appendix 3: data extraction form. Appendix 4: Cochrane risk of bias tool for randomized controlled trials. Appendix 5: GRADE guidelines. Appendix 6: description of Chinese medicine. Appendix 7: characteristics of excluded studies table. [file 5550332.f1.docx]

**Appendix 1. Research Protocol**

**The effectiveness of Traditional Chinese Medicine (TCM) as an adjunct treatment on stable COPD patients: a systematic review and meta-analysis**

Chan KH, PhD^a, b,*^, Tsoi YYS, MPH^c^, McCall M, PhD^b^.

^a^Department for Continuing Education and ^b^Department of Primary Care Health Sciences, The University of Oxford.

^c^Independent Researcher Hong Kong China.

Chan KH: [ka.chan2@kellogg.ox.ac.uk](mailto:ka.chan2@kellogg.ox.ac.uk)

McCall M: [marcy.mccall@phc.ox.ac.uk](mailto:marcy.mccall@phc.ox.ac.uk)

Tsoi YYS: [tsoiyinyuenstella@gmail.com](mailto:tsoiyinyuenstella@gmail.com)

*Correspondence: Chan KH, Room 1603, 16/F, Bell House, Block A, 525-543 Nathan Road, Yau Ma Tei, HKSAR (email: [ka.chan2@kellogg.ox.ac.uk](mailto:ka.chan2@kellogg.ox.ac.uk)).

**Abstract [Protocol]**

**Background**: Traditional Chinese Medicine (TCM), including Chinese herbal medicine (CHM) and acupuncture, exhibits beneficial effects on stable chronic obstructive pulmonary disease (COPD). Yet most of systematic reviews only examined either CHM or acupuncture alone, which is not the usual practice in TCM clinic setting. We design a protocol for evaluating the real clinical effectiveness and safety of TCM by combining CHM and acupuncture.

**Methods**: The following databases are searched from inception to November 2019: PubMed, MEDLINE, EMBASE, Cochrane Central Register of Controlled Trials (CENTRAL), Cochrane Library Database, Chinese National Knowledge Infrastructure (CNKI), WANFANG Database, Chinese Scientific and Technological Periodical Database (VIP) and Chinese Biomedical Database (CBM). Any randomized controlled trials examining either acupuncture or CHM on stable COPD are included. Primary outcomes are lung functions, exacerbations, COPD assessment test, adverse events of any cause, all withdrawals, withdrawals due to adverse events of CHM, withdrawals due to adverse events of acupuncture and withdrawals due to lack of efficacy. Secondary outcomes include quality of life, TCM syndrome score and effective rate, and 6-minute walk distance. Two independent reviewers extract data and assess quality of evidence, and generate meta-analysis and risk of bias by Review Manager program. This protocol strictly follows the Preferred Reporting Item for Systematic Review and Meta-analysis Protocols (PRISMA-P) guidelines.

**Results**: This study provides a robust, all-rounded evaluation of the effectiveness and safety for stable COPD in a real TCM practice setup.

**Conclusion**: The conclusion shows whether TCM is effective and safe in treating stable COPD.

**1 Introduction [Protocol]**

- 1. Background

**1.1.1** Description of the Condition

Chronic Obstructive Pulmonary Disease (COPD) is a common, treatable and preventable disease, which is characterized by chronic respiratory symptoms and airflow limitation owing to airway and/ or alveolar abnormalities caused by persistent exposure to noxious gases or molecules. The major known pathogenesis of COPD is a complex mixture of small airways disease, parenchymal destruction and chronic airway and/ or systemic inflammation.

**1.1.2** Global Burden of COPD

COPD is an important cause of chronic morbidity and mortality in the world, which ranks the fourth in the leading cause of death and is projected to be the third by 2020 [1, 2]. It is a common, preventable and treatable disease but poses an economic burden on the society. COPD patients are usually characterized by persistent respiratory symptoms and airflow limitation. Occasionally they may have acute exacerbation induced by respiratory infection and increase the hospitalization and readmission rate.

Current COPD prevalence data show significant differences among countries, probably because of different diagnostic criteria, survey techniques and analytical methods [3]. The Burden of Obstructive Lung Diseases (BOLD) program has reported the prevalence and risk factors for COPD in people aged ≥ 40 in more than 29 countries, and found that COPD is more common in men than women [4, 5]. Up to now, there are around three million deaths per year [6]. The prevalence of COPD is predicted to rise in the coming 30 years and by 2030 there might be over 4.5 million deaths per year from COPD and comorbidities [7, 8].

**1.1.3** Diagnosis in Conventional Medicine

Diagnosis of COPD is primarily by spirometry which measures patient’s airflow limitation. It is the most widely accepted, easily available and reproducible test of lung function. A ratio of post-bronchodilator forced expiratory volume in first second (FEV_1_)/ forced vital capacity (FVC) < 0.70 confirms the presence of persistent airflow limitation [9]. Different stages of airflow limitation is listed in Table 1. Main symptoms include dyspnea, chronic cough, chronic sputum production, wheezing and chest tightness. But the severity of airflow limitation is weakly correlated with symptoms in clinical context [10] and spirometry itself has a relatively low specificity [11]. So other symptom assessments are required to categorize COPD patients, which commonly include the Modified British Medical Research Council (mMRC) Questionnaire [12] and COPD Assessment Test (CAT^TM^) [13-15].

COPD patients may suffer acute worsening of respiratory symptoms that lead to additional therapy, namely acute exacerbations [16-19]. There are three classifications of exacerbations: mild (short acting bronchodilators (SABDs) only), moderate (SABDs plus antibiotics and/or oral corticosteroids) and severe (hospitalization or visiting emergency room. The best indicator of frequent exacerbations (defined as two or more exacerbations per annum) is a history of earlier treated events [20]. Apart from these tests, physical exercise measurements, like paced shuttle walk test and the unpaced 6-minute walk test are also suggested for monitoring patient health status and predicting prognosis [21-23].

**1.1.4** Conventional pharmacological treatment for stable COPD

For stable COPD, the goals of pharmacological therapy are to reduce symptoms, to reduce the frequency and severity of exacerbations, and to improve health status and exercise tolerance. Apart from smoking cessation and vaccinations, there are two major classes of medications: bronchodilators and anti-inflammatory drugs.

Bronchodilators can increase FEV_1_ and/ or modify other spirometric values and are usually prescribed regularly to prevent or reduce symptoms. Commonly used bronchodilators, mainly short-acting and long-acting beta_2_-agonists (SABA and LABA respectively) and short-acting and long-acting anticholinergics (SAMA and LAMA respectively) are listed in Table 2 [24-27].

- - 1. The Use of Traditional Chinese Medicine for COPD

Traditional Chinese Medicine (TCM) has been using to treat symptoms similar to those in COPD, for instance, cough, sputum or shortness of breath, and has shown beneficial effects for over hundreds of decades. However, there is no such a disease term as COPD in TCM. Instead, COPD patients are classified as having “Fei Zhang” with reference to TCM theory [28, 29]. In normal TCM clinical setting, either Chinese herbal medicine, acupuncture, or the combination of both are used to relieve COPD symptoms, improve lung functions and/ or exercise tolerance [30-33].

TCM is very different from contemporary medicine in both diagnosis and treatment methods. Commonly used TCM treatments include herbal medicinal formula, acupuncture, moxibustion, Tuinai, or the combination of them. In daily TCM healthcare setting, patients with COPD symptoms are often given a set of treatments like acupuncture/ moxibustion, or acupuncture/ medicinal formula. Most RCTs for TCM treatments were conducted only on several acupoints, or a single herb or formulae, which is not similar to the usual TCM practice. This study aims to examine the effectiveness and adverse effects of adding TCM treatments on western medicine in stable COPD, to synthesize the best available data towards recommendations of optimal treatment.

**1.2** Research Objectives

1) The primary objective of this study is to measure the effectiveness of TCM as an adjunct treatment on stable COPD patients in any setting, and the adverse events associated with its use in clinical trials measured by lung function and exacerbation rate. .

2) The secondary objective of this study is to compare the efficacy of either herbal medicine, acupuncture or the combination of both on treating stable COPD patients reflected by TCM syndrome score and health status.

**1.3** PICOS

Population: Patients with stable COPD aged >18 years old, of any sex, education and socio-economic status.

Interventions: Add-on TCM treatment, either herbal medicine, acupuncture or the combination of both, on conventional medicine.

Controls/ Comparators: mainstream pharmacotherapy for managing stable COPD

Outcomes: lung functions as measure by FEV_1_ using spirometry, exacerbation rate, 6-minute walk test, health-related quality of life (QoL)

Study design: double-blind, randomized controlled clinical trials.

1. **Methods [Protocol]**

This review protocol is prepared with reference to the Preferred Reporting Item for Systematic Review and Meta-analysis Protocols (PRISMA-P) [34] and the Cochrane Handbook for Systematic Reviews of Interventions [35], and registered on international prospective register of systematic review (PROSPERO) on 10.12.2019 (registration number: CRD42019161324).

- 1. Inclusion and Exclusion criteria for studies
     1. Types of included studies

Any randomized controlled trials (RCT) with double-blind assessment of patient reported outcomes, of which both patients and assessors are blind to the treatments given, are included. RCTs published in a peer-reviewed journal with full text are requested, and unpublished clinical trials with online results available are included.

- - 1. Types of excluded studies

Abstracts alone, non-randomized trials, case reports, cohort studies, case-control studies, cross-sectional studies, retrospective surveys or chart reviews, editorials, commentaries, and clinical observations are excluded from this systematic review. Other systematic reviews are not included, but the references lists of similar are searched.

- - 1. Types of included participants

We include 1) patients who are 18 year-old or above, regardless of sex, education, race and socio-economic status, and 2) patients are diagnosed with stable COPD according to the diagnostic criteria from the Global Initiative for Chronic Obstructive Lung Disease (GOLD) [36]. Stable COPD patients are defined as patients having mild cough, expectoration and dyspnea.

- - 1. Types of excluded participants

We exclude patients with other diseases such as asthma, tuberculosis, bronchiolitis, congestive heart failure, or other severe complications.

- - 1. Types of interventions

We include any herbal drugs, extracted active ingredients or formula administered orally, of which can be either in a form of TCM granules or boiled soup, and compared to placebo, no treatment, any active comparator or western medicine. We also include any acupuncture treatment, or dry needling, using any acupoint combinations, and compared to placebo, no treatment, any active comparator or conventional medicine. Studies in any healthcare and any global setting are included. Interventions either alone or in combination with each other are included.

- - 1. Types of outcome measures
       1. Primary outcomes

We include the following items as primary outcomes: 1) lung functions by measuring and change in FEV_1_ [37]; 2) exacerbations defined as time-to-first exacerbation or exacerbation rate [36]; 3) COPD assessment test [38]; 4) and adverse events of any cause; 5) all withdrawals; 6) withdrawals due to adverse events of CHM; 7) withdrawals due to adverse events of acupuncture; and 8) withdrawals due to lack of efficacy.

- - - 1. Secondary outcomes

We include quality of life such as sleep patterns, mood and mental health, physical exercise regime on a validated scale; 2) TCM syndrome score and effective rate [39]; and 3) 6-minute walk distance [40] as secondary outcomes.

- - 1. Search strategy

The lead author (KH) design the search strategy and carry out the searches. A broad search strategy will be used to cover all Chinese herbal medicine and acupuncture RCTs to include as many relevant and potentially included trials as possible, from studies inception to November 2019.

- - - 1. Electronic Searches

The following databases will be searched mainly in English and Chinese languages and filtered for humans:

1. PubMed;
2. MEDLINE;
3. EMBASE;
4. Cochrane Central Register of Controlled Trials (CENTRAL);
5. Chinese National Knowledge Infrastructure (CNKI);
6. WANFANG Database;
7. Chinese Scientific and Technological Periodical Database (VIP);
8. Chinese Biomedical Database (CBM);
9. Cochrane Library Database.

The search strategies are tailor-made to each database with a combination of text-words and medical subject headings (MeSH), or an equivalent, and search terms are listed in Table 3.

Moreover, the following online registries will be searched in English and Chinese language and filtered for humans:

1. ClinicalTrials.gov;
2. The *meta*Register of controlled trials (*m*RCT);
3. The World Health Organization (WHO) International Clinical Trials Registry Platform (ICTRP).
   - - 1. Searching other resources

Bibliographies and reference lists of related publications which match with the eligibility criteria will be hand searched, such that we do not miss any important references during the selection process.

- 1. Data collection and analysis
     1. Data extraction and management

Two reviewers (KH and YYS) independently extracted study information and outcome data using a standardized data extraction table for RCTS only [41] that includes title, first author, publication year, country, sample size, age and sex of participants, intervention, treatment duration, follow-up period, outcomes, and adverse events. Extracted data are crosschecked and entered into STATA (version 16). Any disagreement about extracted data will be adjudicated by the third reviewer (MM) and all three reviewers will resolve by discussion and consensus. A sample PRISMA flow chart shows the documentation in Figure 1.

- - 1. Risk of bias assessment

Two authors (KH and YYS) will independently assess the risk of bias for each record using the Cochrane Risk of Bias Tool as reported in the Cochrane Handbook for Systematic Reviews of Interventions [35].

A Risk of Bias table is included as part of each Characteristics of Included Studies table. When facing disagreement about risk of bias, a third reviewer (MM) will adjudicate and all three reviewers will resolve by discussion. Risk of bias is assessed at the individual study level and the risk of bias will also be considered when assessing Grading of Recommendations, Assessment, Development and Evaluation system (GRADE) [42].

These seven domains will be assessed for each included study as outlined by the *Cochrane Handbook for Systematic Reviews of Interventions* [35]:

1. Random sequence generation (examine potential selection bias): studies are assessed for the methods used to generate the allocation sequence;
2. Allocation concealment (examine potential selection bias): studies are assessed for the methods used to conceal allocation to interventions prior to study starts;
3. Blinding of participants and personnel (examine potential performance bias): studies are assessed for methods used to blind the participants and personnel from knowing which intervention a participant will receive;
4. Blinding of outcome assessment (examine potential detection bias): studies are assessed for methods used to blind the outcome assessors from knowing which intervention a participant will receive;
5. Incomplete outcome data (examine potential attrition bias): studies are assessed for the nature, number, and handling of incomplete outcome data;
6. Selective reporting (examine potential reporting bias): studies are assessed whether all planned outcomes are reported in the results;
7. Other bias: studies are assessed for any additional sources of bias as low, unclear or high, and provided rationale.

**2.3.3** Dealing with missing data

Studies not using intention-to-treat (ITT) or unclear are excluded. The ITT pool is calculated as the number of participants who were properly randomized, took at least one dose of the assigned treatment, and done at least one outcome assessment post-baseline [35]. When appropriate, withdrawn or missing patients are regarded as zero improvement [35].

**2.3.4** Assessment of heterogeneity

To evaluate clinical heterogeneity, only studies with similar conditions and treatments are compared to get a clinically useful result [35]. Statistical heterogeneity is assessed visually [42] with the I^2^ statistic and *p* value. If I^2^ is larger than 50%, possible reasons are discussed [35].

- - 1. Data synthesis

The meta-analysis is conducted on extracted data using STATA (version 16) after which results and forest plots are exported into this final report. A fixed-effect model is used for the meta-analysis [35]. When significant clinical heterogeneity is seen, a random-effect model is then used to combine studies [35].

- - 1. Quality of the evidence

GRADE is used to assess the quality of evidence related to each outcome measure and to provide recommendations for clinical practice [35, 43]. A GRADE rating is assigned for each primary and secondary outcome using the four key levels: *high*, *moderate*, *low*, or *very low-quality*, with reasons provided to upgrade or downgrade [43]. Under certain circumstances, the overall GRADE rating might require adjustment. For instance, a study reports very small sample sizes and results are at risk of being down to play of chance [44]. On the other hand, if no data is reported for an outcome, the term ‘no evidence’ or ‘lack of evidence’ can imply there are data and that the results might show no evidence of effect.

- - 1. Subgroup analysis

Subgroup analyses are performed to assess factors like different TCM dosage, forms, and duration of treatment, acupoints used, and measurements of results. Sensitivity analysis is conducted to examine heterogeneity. The effect of methodological quality, sample size, or missing data is also considered. Analysis is repeated after removing methodologically low quality studies.

- - 1. Publication bias

If more than ten studies are selected, the Egger regression test is used to assess funnel plots for any possible publication bias [45].

**2.3.9** Ethical considerations

There are no ethical issues or approvals needed for this type of study as it uses aggregate data already anonymized.

**3 Dissemination [Protocol]**

Results of this systematic review are planned to be shared through publication in a peer-reviewed journal.

**4 Funding [Protocol]**

No specific funding is received for this study. This study is part of the MSc dissertation of the first author (KH CHAN) and is funded by himself. KH CHAN is the lead investigator of this project.

**5 Conflict of interest [Protocol]**

No conflict of interest is reported.

Author contributions

Conceptualization: CHAN KH

Data curation: CHAN KH, TSOI YYS

Formal analysis: CHAN KH

Project administration: CHAN KH

Resources: CHAN KH

Software: CHAN KH

Visualization: CHAN KH

Writing – original draft: CHAN KH

Writing – review & editing: McCall M

**References [Protocol]**

1. Lozano R, Naghavi M, Foreman K, et al. Global and regional mortality from 235 causes of death for 20 age groups in 1990 and 2010: a systematic analysis for the Global Burden of Disease Study 2010. Lancet 2012;280(9859):2095-128.
2. Vos T, Flaxman AD, Naghavi M, et al. Years lived with disability (YLDs) for 1160 sequelae of 289 disease and injuries 1990-2010: a systematic analysis for the Global Burden of Disease Study 2010. Lancet 2012;380(9859):2163-96.
3. Mathers CD, Loncar D. Projections of global mortality and burden of disease from 2002 to 2030. PLoS Med 2006;3(11):e442.
4. BOLD. Burden of Obstructive Lung Disease Initiative Webpage, published by Imperial College London. <http://www.boldstudy.org/> (assessed 1 November 2019).
5. Lamprecht B, McBurnie MA, Vollmer WM, et al. COPD in never smokers: results from the population-based burden of obstructive lung disease study. Chest 2011;139(4):752-63.
6. Global Burden of Disease Study Collaborators. Global, regional, and national age-sex specific all-cause and cause-specific mortality for 240 causes of death, 1990-2013: a systematic analysis for the Global Burden of Disease Study 2013. Lancet 2015;385(9963):117-71.
7. Lopez AD, Shibuya K, Rao C, et al. Chronic obstructive pulmonary disease: current burden and future projections. Eur Respir J 2006;27(2):397-412.
8. World Health Organization. Projections of mortality and causes of death, 2015 and 2030. <http://www.who.int/healthinfo/global_burden_disease/projections/en/> (assessed 1 November 2019).
9. Buist AS, McBurnie MA, Vollmer WM, et al. International variation in the prevalence of COPD (the BOLD Study): a population-based prevalence study. Lancet 2007;370(9589):741-50.
10. Montes de Oca M, Perez-Padilla R, Talamo C, et al. Acute bronchodilator responsiveness in subjects with and without airflow obstruction in five Latin American cities: the PLATINO study. Pulm Pharmacol Ther 2010;23(1):29-35.
11. Jackson H, Hubbard R. Detecting chronic obstructive pulmonary disease using peak flow rate: cross sectional survey. BMJ 2003;327(7416):653-4.
12. Fletcher CM. Standardized questionnaire on respiratory symptoms: a statement prepared and approved by the MRC Committee on the Aetiology of Chronic Bronchitis (MRC breathlessness score). BMJ 1960;2:1662.
13. Miravitlles M, Worth H, Soler Cataluna JJ, et al. Observational study to characterize 24-hour COPD symptoms and their relationship with patient-reported outcomes: results from the ASSESS study. Respir Res 2014;15:122.
14. Jones PW, Harding G, Berry P, Wilund I, Chen WH, Kline Leidy N. Development and first validation of the COPD Assessment Test. Eur Respir J 2009;34(3):648-54.
15. Karloh M, Fleig Mayer A, Maurici R, Pizzichini MM, Jones PW, Pizzichini E. The COPD Assessment Test: What Do We Know So Far?: A Systematic Review and Meta-Analysis About Clinical Outcomes Prediction and Classification of Patients Into GOLD Stages. Chest 2016;149(2):413-25.
16. Hurst JR, Wedzicha JA. What is (and what is not) a COPD exacerbation: thoughts from the new GOLD guidelines. Thorax 2007;62(3):198-9.
17. Wedzicha JA, Seemungal TA. COPD exacerbations: defining their cause and prevention. Lancet 2007;370(9589):786-96.
18. Seemungal TA, Donalson GC, Paul EA, Bestall JC, Jeffries DJ, Wedzicha JA. Effect of exacerbation on quality of life in patients with chronic obstructive pulmonary disease. Am J Respir Crit Care Med 1998;157(5 Pt 1):1418-22.
19. Burge S, Wedzicha JA. COPD exacerbations: definitions and classification. Eur Respir J Suppl 2003;41:46S-53S.
20. Hurst JR, Vestbo J, Anzueto A, et al. Susceptibility to exacerbation in chronic obstructive pulmonary disease. N Engl J Med 2010;363(12):1128-38.
21. Revill SM, Morgan MD, Singh SJ, Williams J, Hardman AE. The endurance shuttle walk: a new field test for the assessment of endurance capacity in chronic obstructive pulmonary disease. Thorax 1999;54(3):213-22.
22. Casanova C, Cote CG, Marin JM, et al. The 6-min walking distance: long-term follow up in patients with COPD. Eur Respir J 2007;29(3):535-40.
23. Puente-Maestu L, Palange P, Casaburi R, et al. Use of exercise testing in the evaluation of interventional efficacy: an official ERS statement. Eur Respir J 2016;47(2):429-60.
24. Sestini P, Renzoni E, Robinson S, Poole P, Ram FS. Short-acting beta 2 agonists for stable chronic obstructive pulmonary disease. Cochrane Database Syst Rev 2002;(4):CD001495.
25. Kew KM, Mavergames C, Walters JA. Long-acting beta2-agonists for chronic obstructive pulmonary disease. Cochrane Database Syst Rev 2013;10(10):CD010177.
26. Han J, Dai L, Zhong N. Indacaterol on dysnea in chronic obstructive pulmonary disease: a systematic review and meta-analysis of randomized placebo-controlled trials. BMC Pulm Med 2013;13:26.
27. Geake JB, Dabscheck EJ, Wood-Bader R, Cates CJ. Indacaterol, a once-daily beta2-agonist, versus twice-daily beta(2)-agonists or placebo for chronic obstructive pulmonary disease. Cochrane Database Syst Rev 2015;1:CD010139.
28. <<Treatise on exogenous febrile disease and synopsis of golden chamber>>. Chapter 7 (Chinese).
29. Professional Committee of Pulmonary Diseases of Internal Medicine Branch of Chinese Medical Association Traditional Chinse medicine diagnosis and treatment guide for chronic obstructive pulmonary disease (Chinese). Zhong Yi Za Zhi 2012;53:80-4.
30. Guo S, Sun ZT, Liu ES, et al. Effect of Bufei granule on stable chronic obstructive pulmonary disease: a randomized, double blinded, placebo-controlled, and multicenter clinical study. J Tradit Chin Med 2014;43:437-44.
31. Hong M, Hong C, Chen H, et al. Effects of the Chinese herb formula Yufeining on stable chronic obstructive pulmonary disease: a randomized, double-blind, placebo-controlled trial. Medicine (Baltimore) 2018;97:1-7.
32. Liu Y, Huang Y, Zhao C, et al. Salvia miltiorrhiza injection on pulmonary heart disease: a systematic review and meta-analysis. Am J Chin Med 2014;42:1315-31.
33. Shergis JL, Liu S, Chen X, et al. Dang shen [Codonopsis pilosula (Franch.) Nannf] herbal formulae for chronic obstructive pulmonary disease: a systematic review and meta-analysis. Phytother Res 2015;29:167-86.
34. Moher D, Shamseer L, Clarke M, et al. Preferred reporting items for systematic review and meta-analysis protocols (PRISMA-P) 2015 statement. Syst Rev 2015;4:1-9.
35. Higgins, J. P. T. & Thomas J. (editors) 2019. Cochrane Handbook for Systematic Reviews of Interventions, Version 6. The Cochrane Collaboration. Available at: <https://training.cochrane.org/handbook/current>.
36. Global Initiative for Chronic Obstructive Lung Disease (GOLD). Global strategy for the diagnosis, management and prevention of COPD. Updated 2019. Available at: [www.goldcopd.com](http://www.goldcopd.com). (accessed 15 October 2019).
37. American Thoracic Society Standardization of spirometry, 1994 update. Am J Respir Crit Care Med 1995;152:1107-36.
38. Raghavan N, Lam YM, Webb KA, et al. Components of the COPD Assessment Test (CAT) associated with a diagnosis of COPD in a random population sample. COPD 2012;9:175-83.
39. Zheng XY. Guiding Principles for Clinical Research of New TCM Drugs (Chinse). 2002;China Medical Science and Technology Press.
40. ATS Committee on Proficiency Standards for Clinical Pulmonary Function Laboratories ATS statement: guidelines for the six-minute walk test. Am J Respir Crit Care Med 2002;166:111-7.
41. Data collection form for intervention reviews: RCTs only. Cochrane Developmental, Psychosocial and Learning Problems. Updated 2014. Available at: <https://dplp.cochrane.org/data-extraction-forms>. (accessed 5 December 2019).
42. L’Abbe KA, Detsky AS & O’Rourke K. Meta-analysis in clinical research. Annals of Internal Medicine 1987;107:224-233.
43. Guyatt GH, et al. GRADE: an emerging consensus on rating quality of evidence and strength of recommendations. BMJ 2008;336:924-6.
44. Guyatt G, et al. Making an overall rating of the confidence in effect estimates for a single outcome and for all outcomes. Journal of Clinical Epidemiology 2013a;66(2):151-157.
45. Egger M, Smith GD, Schneider M, et al. Bias in meta-analysis detected by a simple, graphical test. BMJ 1997;315:629-34.

**Figure 1 PRISMA study flowchart of search results [Protocol]**

Additional records identified from other sources

(n = )

Records identified from nine databases searched

(n = )

Identification

Records after removing duplicates

(n = )

Screening

Records excluded (n = )

Not related to COPD

Not related to CHM

Not related to Acupuncture

Not related to human

Not clinical trials, etc.

Records screened

(n = 　)

Full text articles assessed for eligibility

(n = 　)

Full text excluded (n = )

Non RCTs

Case studies

Cohort studies

Surveys, etc.

Eligibility

Studies included in quantitative synthesis (meta-analysis)

(n = 　)

Studies included in quantitative synthesis

(n = 　)

Included

**Table 1. Severity of airflow limitation. [Protocol]**

| Grade | FEV_1_ (% predicted) |
| --- | --- |
| GOLD 1 | ≥ 80 |
| GOLD 2 | 50-79 |
| GOLD 3 | 30-49 |
| GOLD 4 | < 30 |

**Table 2. Bronchodilators used in stable COPD. [Protocol]**

| **Drug Classification** | **Generic Drug Name** |
| --- | --- |
| **Beta_2_-agonists** | |
| **SABA** | Fenoterol, Levalbuterol, Salbutamol, Terbutaline |
| **LABA** | Arformoterol, Formoterol, Indacaterol, Olodaterol, Sameterol |
| **Anticholinergics** | |
| **SAMA** | Ipratropim bromide, Oxitropium bromide |
| **LAMA** | Aclidinium bromide, Glycopyrronium bromide, Tiotropium, Umeclidinium |
| **Methylxanthines** | Aminophylline, Theophylline |
| **Phosphodiesterase-4 inhibitors** | Roflumilast |
| **Mucolytic agents** | Erdosteine |

**Table 3. Search terms used in PubMed. [Protocol]**

| Number | Search terms |
| --- | --- |
| 1 | Randomized controlled trial |
| 2 | RCT |
| 3 | Randomized |
| 4 | Randomly |
| 5 | Trial |
| 6 | Groups |
| 7 | Controlled clinical trial |
| 8 | 1 or 2-7 |
| 9 | Chronic obstructive pulmonary disease |
| 10 | COPD |
| 11 | Chronic obstructive airway disease |
| 12 | Chronic obstructive respiratory disease |
| 13 | Chronic bronchitis |
| 14 | Emphysema |
| 15 | Chronic airflow obstruction |
| 16 | 9 or 10-15 |
| 17 | Chinese Medicine |
| 18 | Chinese Herbal Medicine |
| 19 | CHM |
| 20 | Traditional Chinese Medicine |
| 21 | TCM |
| 22 | Traditional medicine |
| 23 | Herb* |
| 24 | Herb* medicine |
| 25 | Plant medicine |
| 26 | Herb formula |
| 27 | Herb decoction |
| 28 | 17 or 18-27 |
| 29 | Acupuncture |
| 30 | Acupoint* |
| 31 | Needling |
| 32 | Dry Needling |
| 33 | 29, 30-32 |
| 34 | 8 and 16 and 28 and 33 |

**Appendix 2. PRISMA statement reporting standards checklist.**

| Section/topic | Number | Checklist item |
| --- | --- | --- |
| **TITLE** |  |  |
| Title | 1 | Identify the report as a systematic review, meta-analysis, or both. |
| **ABSTRACT** |  |  |
| Structured summary | 2 | Provide a structured summary including, as applicable: background; objectives; data sources; study eligibility criteria, participants, and interventions; study appraisal and synthesis methods; results; limitations; conclusions and implications of key findings; systematic review registration number. |
| **INTRODUCTION** |  |  |
| Rationale | 3 | Describe the rationale for the review in the context of what is already known. |
| Objectives | 4 | Provide an explicit statement of questions being addressed with reference to participants, interventions, comparisons, outcomes, and study design (PICOS). |
| **METHODS** |  |  |
| Protocol and registration | 5 | Indicate if a review protocol exists, if and where it can be accessed (e.g., Web address), and, if available, provide registration information including registration number. |
| Eligibility criteria | 6 | Specify study characteristics (e.g., PICOS, length of follow-up) and report characteristics (e.g., years considered, language, publication status) used as criteria for eligibility, giving rationale. |
| Information sources | 7 | Describe all information sources (e.g., databases with dates of coverage, contact with study authors to identify additional studies) in the search and date last searched. |
| Search | 8 | Present full electronic search strategy for at least one database, including any limits used, such that it could be repeated. |
| Study selection | 9 | State the process for selecting studies (i.e., screening, eligibility, included in systematic review, and, if applicable, included in the meta-analysis). |
| Data collection process | 10 | Describe method of data extraction from reports (e.g., piloted forms, independently, in duplicate) and any processes for obtaining and confirming data from investigators. |
| Data items | 11 | List and define all variables for which data were sought (e.g., PICOS, funding sources) and any assumptions and simplifications made. |
| Risk of bias in individual studies | 12 | Describe methods used for assessing risk of bias of individual studies (including specification of whether this was done at the study or outcome level), and how this information is to be used in any data synthesis. |
| Summary measures | 13 | State the principal summary measures (e.g., risk ratio, difference in means). |
| Synthesis of results | 14 | Describe the methods of handling data and combining results of studies, if done, including measures of consistency (e.g., I2) for each metaanalysis. |
| Risk of bias across studies | 15 | Specify any assessment of risk of bias that may affect the cumulative evidence (e.g., publication bias, selective reporting within studies). |
| Additional analyses | 16 | Describe methods of additional analyses (e.g., sensitivity or subgroup analyses, meta-regression), if done, indicating which were pre-specified. |
| **RESULTS** |  |  |
| Study selection | 17 | Give numbers of studies screened, assessed for eligibility, and included in the review, with reasons for exclusions at each stage, ideally with a flow diagram |
| Study characteristics | 18 | For each study, present characteristics for which data were extracted (e.g., study size, PICOS, follow-up period) and provide the citations. |
| Risk of bias within studies | 19 | Present data on risk of bias of each study and, if available, any outcome level assessment (see item 12). |
| Results of individual studies | 20 | For all outcomes considered (benefits or harms), present, for each study: (a) simple summary data for each intervention group (b) effect estimates and confidence intervals, ideally with a forest plot. |
| Synthesis of results | 21 | Present results of each meta-analysis done, including confidence intervals and measures of consistency. |
| Risk of bias across studies | 22 | Present results of any assessment of risk of bias across studies (see Item 15). |
| Additional analysis | 23 | Give results of additional analyses, if done (e.g., sensitivity or subgroup analyses, meta-regression [see Item 16]). |
| **DISCUSSION** |  |  |
| Summary of evidence | 24 | Summarize the main findings including the strength of evidence for each main outcome; consider their relevance to key groups (e.g., healthcare providers, users, and policy makers). |
| Limitations | 25 | Discuss limitations at study and outcome level (e.g., risk of bias), and at review-level (e.g., incomplete retrieval of identified research, reporting bias). |
| Conclusions | 26 | Provide a general interpretation of the results in the context of other evidence, and implications for future research. |
| **FUNDING** |  |  |
| Funding | 27 | Describe sources of funding for the systematic review and other support (e.g., supply of data); role of funders for the systematic review. |
| **I2:** I squared; **PICOS:** population, intervention, comparison, outcomes, study design. | | |

**Appendix 3. Data extraction form.**

| Study name | Study 1 |
| --- | --- |
| Methods | Allocation:  Blinding:  Controlled:  Arms:  Centers:  Study duration: |
| Participants | Inclusion criteria:  Exclusion criteria:  Sample size (Intervention/ control):  Ages:  Course of years:  Gender:  Treatment duration:  Baseline difference:  Quality control: |
| Interventions | Composition of drugs:  Dosage: |
| Outcome | Primary outcomes:  Secondary outcomes: |
| RoB: Randomization sequence generation | Low/ Unclear/ High  Quote:  Comment: |
| RoB: Allocation concealment | Low/ Unclear/ High  Quote:  Comment: |
| RoB: Blinding of participants and personnel | Low/ Unclear/ High  Quote:  Comment: |
| RoB: Blinding of outcome assessors | Low/ Unclear/ High  Quote:  Comment: |
| RoB: Incomplete outcome data | Low/ Unclear/ High  Quote:  Comment: |
| RoB: Selective reporting | Low/ Unclear/ High  Quote:  Comment: |
| RoB: Other | Low/ Unclear/ High  Quote:  Comment: |
| P1: Change in FEV_1_ |  |
| P2: Exacerbation rate |  |
| P3: COPD assessment test |  |
| P4: Adverse events of any cause |  |
| P5: All withdrawals |  |
| P6: Withdrawals due to adverse event of CHM |  |
| P7: Withdrawals due to adverse events of acupuncture |  |
| P8: Withdrawals due to lack of efficacy |  |
| S1: Quality of life |  |
| S2: TCM syndrome and effective rate |  |
| S3: 6-minute walk test |  |
| RoB: risk of bias domain; P1-8: primary outcomes; S1-3: secondary outcomes; FEV_1_: forced expiratory volume in 1s; TCM: Traditional Chinese Medicine. | |

**Appendix 4. Cochrane risk of bias tool for randomized controlled trials.**

| **Domain** | **Support for judgement** | **Review authors’ judgement** |
| --- | --- | --- |
| **Selection bias** |  |  |
| Random sequence generation | Describe the method used to generate the allocation sequence in sufficient detail to allow an assessment of whether it should produce comparable groups. | Selection bias (biased allocation to interventions) due to inadequate generation of a randomised sequence. |
| Allocation concealment | Describe the method used to conceal the allocation sequence in sufficient detail to determine whether intervention allocations could have been foreseen in advance of, or during, enrolment. | Selection bias (biased allocation to interventions) due to inadequate concealment of allocations prior to assignment. |
| **Performance bias** |  |  |
| Blinding of participants and personnel. | Describe all measures used, if any, to blind study participants and personnel from knowledge of which intervention a participant received. Provide any information relating to whether the intended blinding was effective. | Performance bias due to knowledge of the allocated interventions by participants and personnel during the study. |
| **Detection bias** |  |  |
| Blinding of outcome assessment. | Describe all measures used, if any, to blind outcome assessors from knowledge of which intervention a participant received. Provide any information relating to whether the intended blinding was effective | Detection bias due to knowledge of the allocated interventions by outcome assessors. |
| **Attrition bias** |  |  |
| Incomplete outcome data. | Describe the completeness of outcome data for each main outcome, including attrition and exclusions from the analysis. State whether attrition and exclusions were reported, the numbers in each intervention group (compared with total randomized participants), reasons for attrition/exclusions where reported, and any re-inclusions in analyses performed by the review authors. | Attrition bias due to amount, nature or handling of incomplete outcome data. |
| **Reporting bias** |  |  |
| Selective reporting | State how the possibility of selective outcome reporting was examined by the review authors, and what was found. | Reporting bias due to selective outcome reporting. |
| **Other bias** |  |  |
| Other sources of bias | State any important concerns about bias not addressed in the other domains in the tool. If particular questions/entries were pre-specified in the review’s protocol, responses should be provided for each question/entry. | Bias due to problems not covered elsewhere in the table. |

**Appendix 5. GRADE guidelines.**

| High quality | We are very confident that the true effect lies close to that of the estimate of effect. | Further research is very unlikely to change our confidence in the estimate of effect. |
| --- | --- | --- |
| Moderate quality | We are moderately confident in the effect estimate; the true effect is likely to be close to the estimate of effect, but there is a possibility that it is substantially different. | Further research is likely to have an important impact on our confidence in the estimate of effect and may change the estimate. |
| Low quality | Our confidence in the effect estimate is limited; the true effect may be substantially different from the estimate of the effect. | Further research is very likely to have an important impact on our confidence in the estimate of effect and is likely to change the estimate. |
| Very low quality | We have very little confidence in the effect estimate; the true effect is likely to be substantially different from the estimate of effect. | Any estimate of effect is very uncertain. |
| No evidence to support or refute (Guyatt, et al., 2013a) | There is no data to calculate an effect estimate. | No judgement can be made to support or refute the intervention. |
|  | Reasons to downgrade | Reasons to upgrade |
|  | • serious (-1) or very serious (-2) limitation  to study quality;  • important inconsistency (-1);  • some (-1) or major (-2) uncertainty about directness;  • imprecise or sparse data (-1);  • high probability of reporting bias (-1). | • strong evidence of association – significant relative risk of > 2 (< 0.5) based on consistent evidence from two or more observational studies, with no plausible confounders (+1);  • very strong evidence of association – significant relative risk of > 5 (< 0.2) based on direct evidence with no major threats to validity (+2);  • evidence of a dose response gradient (+1);  • all plausible confounders would have reduced the effect (+1). |
| **+:** plus; **-:** minus; **>:** greater than; **<:** less than |  |  |

**Appendix 6. Description of Chinese Medicine.**

| Study | | Formula (Short form) | Formula | Components | Dosage and frequency |
| --- | --- | --- | --- | --- | --- |
| WANG | 2019 | MSZYQD | Modified San Zi Yang Qin Decocton | *Huangqi 30g, Zisuzi 12g, Baijiezi 9g, Laifuzi 9g, Fuling 12g, Baizhu 15g, Chenpi 9g, Wuweizi 10g, Gancao 6g* | 1 dosage per day, intake twice a day |
| ZHU | 2019 | YQYYTBLMZY | Yi Qi Yang Yin Tong Bu Luo Mai Zhong Yao | *Huangqi 15g, Xiyangshen 10g, Guaizhi 15g, Jiegeng 10g, Tubiechong 6g, Fangfeng 10g, Chaihu 10g, Shengma 5g, Chenpi 10g, Danggui 10g, Quanxie 3g, Wugong 3, Zhigancao 6g* | 1 dosage per day, intake twice a day |
| CHEN | 2019 | MXBFD | Mai Xing Bu Fei Decoction | *Maidong 20g, Xingren10g, Dangshen 20g, Huangqi 20g, Shoudihuang 15g, Wuweizi 10g, Ziyuan 10g, Sangbaipi 10g, Danshen 15g, Zhigancao 6g* | 1 dosage per day, intake twice a day |
| ZHOU | 2019 | BSNQG | Bu Shen Na Qi Granule | *Yinyanghuo 6g, Bajitian 6g, Shechuangzi 3g* | 1 dosage per day, intake twice a day |
| LIU | 2019 | BFHXD | Bu Fei Huo Xue Decoction | *Shoudihuang 20g, Danggui 10g, Yinyanghuo 10g, Huangqi 30g, Xiyangshen 10g, Huangjing 20g, Fabanxia 10g, Huajuhong 15g, Fuling 15g, Wuweizi 6g, Zhigancao 6g, Dilong 10g, Honghua 6g, Chuanqiong 15g, Chaolaifuzi 15g, Taoren 10g* | 1 dosage per day, intake twice a day |
| ZHANG | 2019 | MSZJQD | Modified Su Zi Jiang Qi Decoction | *Zisuzi 12g, Banxia 9g, Houpu 15g, Qianhu 9g, Zisuye 10g, Xingren 10g, Baizhu 15g, Rougui 9g, Yizhiren 10g, Danggui 15g, Quanxie 6g, Dilong 15g, Shengjiang 3pcs, Dazao 3pcs, Gancao 6g* | 1 dosage per day, intake twice a day |
| ZHANG | 2019 | SMZYYFD | Self-made Zi Yin Yang Fei Decoction | *Huangqi 15g, Maidong 10g, Dangshen 10g, Baihe 10g, Zhimu 10g, Sangbaipi 10g, Wuweizi 6g, Zhebeimu 10g, Fuling 15g, Zisuzi 10g, Kuandonghua 10g, Kuxingren 6g, Gancao 6g* | 1 dosage per day, intake twice a day |
| LIN | 2019 | YSBFG | Yi Shen Be Fei Granule | *Ziheche 100g, Buguzhi 100g, Gejie 2 pairs, Shenghuangqi 120g, Fangfeng 80g, Baihsu 100g, Xiyangshen 70g, Sanqifen 70g, (Chuan)beimu 60g, Dilong 100g* | 1 dosage per day, intake twice a day |
| WANG | 2019 | SMQFWSHTD | Self-made Qing Fei Wen Shen Hua Tan Decoction | *Qingtiankui, Shoufuzi, Fabanxia, Yuxingcao, Zhimahuang, Kuandonghua, Wuweizi, Baijiezi, Shixin* | 1 dosage per day, intake twice a day |
| JIN | 2019 | SLBZDAAS | Shen Ling Bai Zhu Decoction Add And Subtract | *Dangshen 20g, Baizhu 10g, Fuling 15g, Chenpi 10g, Baibiandou 15g, Lianzirou 15g, Huaishanyao 30g, Jiegeng 10g, Yiyiren 20g, Sharen 10g, Danshen 20g, Gancao 6g.* | 1 dosage per day, intake twice a day |
| LI | 2019 | FZHTQYM | Fu Zheng Hua Tan Qu Yu Method | *Fuzi 40g, Shengjiang 30g, Huangqi 30g, Dangshen 30g, Fuling 20g, Baizhu 20g, Chishao 30g, Gualouke 30g, Ziyuan 15g, Chenpi 15g, Honghua 10g, Taoren 10g, Zhike 10g, Zhigancao 5g* | 1 dosage per day, intake twice a day |
| LIANG | 2019 | GBQFD | Gu Ben Qing Fei Decoction | *Dangshen 20, Huangqi 20g, Chenpi 15g, Fabanxia 15g, Baizhu 15g, Fuling 15g, Shanyao 10g, Wuweizi 10g, Zhimahuang 6g, Baibiandou 10g, Xingren 10g, Yiyiren 10g, Gancao 6g* | 1 dosage per day, intake twice a day |
| WANG | 2019 | SLBZDAAS | Shen Ling Bai Zhu Decoction Add And Subtract | *Dangshen 20g, Baizhu 10g, Fuling 15g, Chenpi 10g, Baibiandou 15g, Lianzirou 15g, Huaishanyao 30g, Jiegeng 10g, Yiyiren 20g, Sharen 10g, Danshen 20g, Gancao 6g.* | 1 dosage per day, intake twice a day |
| CHEN | 2019 | SQBFD | Shen Qi Bu Fei Decoction | *Huangqi 30g, Danshen 30g, Sangbaipi 30g, Ziyuan 15g, Buguzhi 15g, dangshen 15g, Baibu 15g* | 1 dosage per day, intake twice a day |
| HE | 2019 | SQBFD | Shen Qi Bu Fei Decoction | *Huangqi 30g, Dangshen 15g, Danshen 30g, Baibu 15g, Sangbaipi 30g, Ziyuan 15g* | 1 dosage per day, intake twice a day |
| YUN | 2019 | SQGBD | Shen Qi Gu Ben Decoction | *Dangshen 10g, Shoudihuang 10g, Chenpi 10g, Buguzhi 10g, Chuanbeimu 10g, Chuanqiong 10g, Hutuiziye 10g, Huangqi 15g, Tusizi 15g, Gancao 5g* | 1 dosage per day, intake twice a day |
| ZENG | 2019 | LWBQG | Liu Wei Bu Qi Granule | *Zhihuangqi 20g, Renshen 10g, Yuzhu 10g, Chenpi 6g, Yizhiren 10g, Rougui 3g* | 1 dosage per day, intake three times a day |
| KE | 2019 | JPYFD | Jian Pi Yi Fei Decoction | *Huangqi 20g, Dangshen 10g, Fuling 12g, Chaobaizhu 12g, Maidong 10g, fangfeng 10g, Chenpi 10g, Zhigancao 6g* | 1 dosage per day, intake twice a day |
| FENG | 2018 | QTJFD | Qu Tan Jiu Fei Decoction | *Mahuang 2g, Baijiezi 3g, Jiegeng 3g, Chantui 3g, Gancao 3g, Suzi 3g, Zhuru 5g, Gualoupi 5g, Xingren 5g, Shigao 5g, Jiangchan 5g, Lugen 5g, Huangqin 5g, Pugongying 5g* | 1 dosage per day, intake twice a day |
| HUANG | 2018 | JSLJD + THSWD | Jin Shui Liu Jun Decoction + Tao Hua Si Wu Decoction | *Shoudi 15g, Danggui 10g, Chenpi 12g, Banxia 9g, Fuling 15g, Zhigancao 5g, Huangqi 20g, Dangshen 15g, Taoren 15g, Honghua 5g, Baishao 12g, Chuanqiong 10g* | 1 dosage per day, intake twice a day |
| XU | 2018 | BFHXD | Bu Fei Huo Xue Decoction | *Gancao 10g, Zhebeimu 10g, Baiguo 10g, Zisuzi 10g, Chishao 15g, Quangualou 20g, Huangqi 45g, Chuanqiong 10g, Danshen 30g* | 1 dosage per day, intake twice a day |
| LIU | 2018 | BFHXD | Bu Fei Huo Xue Decoction | *Sangbaipi 30g, Danshen 30g, Baizhu 20g, Quangualou 20g, Huangjing 20g, Huangqi 45g, Chuanqiong 15g, Chenpi 15g, Baiguo 15g, Fuling 15g, Dangshen 15g, Chishao 15g, Buguzhi 15g, Beimu 15g, Danggui 10g, Zisuzi 10g, Dilong 10g, Taoren 10g, Gancao 10g, Banxia 10g, Fangfeng 10g* | 1 dosage per day, intake twice a day |
| KANG | 2018 | BZYQD | Bu Fei Yi Qi Decoction | *Chenpi 10g, Danggui 10g, Chaihu 10g, Shengma 10g, Baizhu 10g, Xidangshen 20g, Shenghuangqi 25g, Zhigancao 6g* | 1 dosage per day, intake twice a day |
| WANG | 2018 | YQBFD | Yi Qi Bu Fei Decoction | *Huangqi 30g, Maidong 15g, Beishashen 10g, Danshen 15g, Xingren 10g, Ziyuan 10g, Sangbaipi 10g, Zhigancao 5g* | 1 dosage per day, intake twice a day |
| LU | 2018 | LJZD | Liu Jun Zi Decoction | *Fabanxia 9g, Dangshen 18g, Gancao 6g, Baizhu 18g, Chenpi 6g, Fuling 18g* | 1 dosage per day, intake twice a day |
| YANG | 2018 | FFGSD | Fu Fei Gu Shen Decoction | *Bajitian 9g, Ziheche 9g, Buguzhi 9g, Shanyurou 9g, Shoudihuang 15g, Xiyangshenpian 9g, Chaobaizhu 6g, Fuling 9g, Ganjiang 5g, Zhichuanqiong 5g, Maidong 10g, Taoren 6g, Zisuzi 5g, Chaotinglizi 15g, Shenmuli 15g, honghua 6g, Chaodilong 6g, Gancao 5g* | 1 dosage per day, intake twice a day |
| LI | 2018 | MZFWDD | Man Zu Fei Wen Ding Decoction | *Huangqi 30g, Dangshen 20g, Nanshashen 15g, Buguzhi 12g, Bajitian 8g, Danggui 15g, Beishashen 15g, Baizhu 20g* | 1 dosage per day, intake twice a day |
| YANG | 2018 | PKHTD | Ping Ke Hua Tan Decoction | *Banxia 10g, Chenpi 5g, Cangzhu 10g, Chuanhoupu 5g, Baizhu 10g, Fuling 5g, Gancaopian 5g.* | 1 dosage per day, intake twice a day |
| GAO | 2018 | SGDHP | Shen Ge Di Huang Pill | *Rensheng 100g, Gejie 40g, Shoudihuang 200g, Buguzhi 200g, Yinyanghuo 150g, Fangfeng 150g, Huangqi 300g, Xingren 100g, Chuanbeimu 60g, Danggui 150g, Danshen 200g, Gancao 100g* | 1 dosage per day, intake three times a day |
| ZHANG | 2018 | SGBFD | Shen Ge Bu Fei Decoction | *Dangshen 30g, Huangqi 30g, Shoudi 15g, Wuweizi 9g, Kuandonghua 9g, Sangbaipi 18g* | 1 dosage per day, intake twice a day |
| WU | 2018 | SLBZD | Shen Ling Bai Zhu Decoction | *Dangshen 20g, Fuling 20g, Baizhu 10g, Baibiandou 30g, Chenpi 5g, Shanyao 30g, Lianzirou 15, Yiyiren 30g, Sharen 6g, Jiegeng 6g, Gancao 6g* | 1 dosage per day, intake twice a day |
| LU | 2018 | SQBFD | Shen Qi Bu Fei Decoction | *Huangqi 30g, Dangshen 15g, Danshen 15g, Sangbaipi 15g, Chenpi 6g, Kuandonghua 12g, Ziyuan 12g, Buguzhi 12g* | 1 dosage per day, intake three times a day |
| FENG | 2018 | SQBFD | Shen Qi Bu Fei Decoction | *Huangqi 30g, Dangshen 15g, Danshen 15g, Sangbaipi 15g, Chenpi 6g, Kuandonghua 12g, Ziyuan 12g, Buguzhi 12g* |  |
| YUE | 2018 | SQBFD | Shen Qi Bu Fei Decoction | *Dangshen 20g, Huangqi 20g, Shoudihuang 15g, Buguzhi 15g, Xianlingpi 15g, Huangjing 15g, Chuangong 15g, Danshen 15g, Wuweizi 15g, Ziyuan 15g, Kuandonghua 15g, Zisuzi 15g, Qingbanxia 10g, Dilong 10g, Gancao 5g* | 1 dosage per day, intake twice a day |
| XU | 2018 | MBXXXD | Modified Ban Xia Xie Xin Decoction | *Fabanxia 10g, Huangqi 10g, Ganjiang 10g, Dangshen 20g, Huanglian 3g, Zhigancao 10g, Dazao 10g, Kuxingren 10g, Gancao 10g, Paodilong 10g, Jianghoupu 10g, Zhimahuang 9g.* | 1 dosage per day, intake once a day |
| TU | 2018 | MLJZD | Modified Liu Jun Zi Decoction | *Dangshen 30g, Fuling 10g, Baizhu 9g, Chenpi 9g, Fabanxia 9g, Gancao 3g, Huangqi 30g, Fangfeng 9g, Danshen 10g* | 1 dosage per day, intake twice a day |
| LI | 2018 | YFBJD | Yang Fei Bu Jin Decoction | *Rensheng 5g, Huangqi 15g, Shoudihuang 15, Ziyuan 10g, Baibu 10g, Xingren 10g, Taoren 10g, Wuweizi 10g, Dilong 10g, Danshen 20g* | 1 dosage per day, intake twice a day |
| DAI | 2017 | SSSQD | San Sang Shen Qi Decoction | *Sangbaipi 10g, Sangjisheng 10g, Sangshenzi 20g, Buguzhi 10g, Huangjing 10g, Wuweizi 6g, Pingdimu 10g, Gonglaoye 10g, Zisugeng 20g, Eguanshi 10g, Kunbu 10g, Fangji 10g* | 1 dosage per day, intake twice a day |
| ZHOU | 2017 | MZFWDF | Man Zu Fei Wen Ding Decoction | *Guizhi 10g, Baizhu 10g, Chuanqiong 10g, Dangshen 10g, Fabanxia 15g, Fuling 10g, Shoudi 30g, Danggui 15g, Mahuang 5g, Shigao 15g* | 1 dosage per day, intake twice a day |
| LIU | 2017 | ZYZYF | Zhong Yao Zi Yi Fang | *Chuanbei 10g, Danggui 10g, Dahuang 10g, Taoren 20g, Yiyiren 20g, Danshen 20g, Liujinu 20g, Yuxingcao 20g* | 1 dosage per day, intake twice a day |
| YU | 2017 | BZYQD | Bu Zhong Yi Qi Decoction | *Gancao 6g, Shenghuangqi 25g, Chenpi 10g, Xidangshen 20g, Danggui 10g, Baizhu 10g, Chaihu 10g, Shengma 10g* | 1 dosage per day, intake twice a day |
| ZHANG | 2017 | SZYQD | San Zi Yang Qin Decoction | *Zisuzi 10g, Baijiezi 6g, Chaolaifuzi 10g, Huaishanyao 60g, Xuanshen 30g, Chenpi 6g, Tinglizi 10g, Dazao 10g* | 1 dosage per day, intake twice a day |
| KONG | 2017 | BFFCD | Bu Fei Fang Chuan Decoction | *Ziyuan 10g, Renshen 10g, Wuweizi 5g, Shoudihuang 20g, Huangqi 30g, Sangbaipi 15g* | 1 dosage per day, intake twice a day |
| WANG | 2017 | ILANKM | Invigorating Lung And Nourishing Kidney Method | *Renshen 12g, Huangqi 12g, Gejie 1 pair, Baizhu 12g, Fangfeng 6g, Fuling 10g, Kuxingren 10g, Chuanbeimu 6g, Sangbaipi 10g, Zhimu 10g, Shenggancao 6g* | 1 dosage per day, intake twice a day |
| WANG | 2017 | BFHXD | Bu Fei Huo Xue Decoction | *Shoudihuang 30g, Huangqi 30g, Baizhu 30g, Yinyanghuo 15g, Fuling 15g, Danggui 15g, Danshen 15g, Dilong 15g, Chuanqiong 15g, Chaolaifuzi 15g, honghua 6g, Taoren 6g, Wuweizi 6g, Gancao 5g* | 1 dosage per day, intake twice a day |
| ZHAO | 2017 | BFD | Bu Fei Decoction | *Renshen 10g, Huangqi 12g, Guiban 15g, Shoudihuang 20g, Taoren 10g, Zhigancao 10g, Ziyuan 20g, Sangbaipi 20g* | 1 dosage per day, intake twice a day |
| GONG | 2017 | BFJPYSD | Bu Fei Jian Pi Yi Shen Decoction | *Renshen 12g, Shenghuangqi 12g, Fangfeng 12g, Fuling 12g, Baizhu 12g, Gejie 9g, Wuweizi 9g, Hutaorou 9g, Zhigancao 15g* | 1 dosage per day, intake twice a day |
| DONG | 2017 | GJDCP | Ge Jie Ding Chuan pill |  | 1 dosage per day, intake twice a day |
| ZHOU | 2017 | SMYFPCD | Self-made Yi Fei Ping Chuan Decoction | *Taizishen 15g, Baizhu 10g, Huangqi 10g, Fuling 10g, Banxia 10g, Chenpi 10g, Mahuang 12g, Huaishanyao 15g, Jiegeng 9g, Sangbaipi 10g, Wuweizi 10g, Danggui 6g, Danshen 10g, Gancao 6g* | 1 dosage per day, intake twice a day |
| XIAO | 2017 | SMWBPSD | Self-made Wen Bu Pi Shen Decoction | *Dangshen 15g, Baishao 12g, Baizhu 12g, Tusizi 12g, Fuling 12g, Dilong 12g, Xianlingpi 12g, Banxia 12g, Danshen 30g, Ganjiang 6g, Zhigancao 3g* | 1 dosage per day, intake twice a day |
| LI | 2017 | SMYYQFD | Self-made Yang Yin Qing Fei Decoction | *Shengmahuang 20g, Maidong 12g, Beimu 12g, Xuanshen 12g, Bohe 12g, Chaobaishao 8g, Huangqi 8g, Danpi 8g, Shenggancao 6g* | 1 dosage per day, intake once a day |
| WEN | 2017 | FLK | Fei Lao Kang |  | 1 dosage per day, intake twice a day |
| YOU | 2017 | YQHTQYTLD | Yi Qi Hua Tan Qu Yu Tong Luo Decoction | *Biejia 20g, Huangqi 30g, Guangdilong 10g, Chishao 15g, Shuizhi 3g, Zhuyazao 3g, Danggui 15g, Chuanqiong 15g, Ziyuan 10g, Fuling 15g* | 1 dosage per day, intake twice a day |
| LU | 2017 | WBFSM | Wen Bu Fei Shen Method |  | 1 dosage per day, intake twice a day |
| YANG | 2017 | FFGSD | Fu Fei Gu Shen Decoction | *Bajitian 9g, Ziheche 9g, Buguzhi 9g, Shanzhuyu 9g, Shoudihuang 15g, Xiyangshenpian 9g, Chaobaizhu 6g, Fuling 9g, Ganjiang 5g, Zhichuanqiong 5g, Maidong 10g, taoren 10g, Zisuzi 5g, Chaotinglizi 15g, Shengmuli 15g, Honghua 6g, Chaodilong 6g, Gancao 5g* | 1 dosage per day, intake twice a day |
| LU | 2017 | PCGBD | Ping Chuan Gu Ben Decoction | *Shenghuangqi 60g, Dangshen 20g, Zisuzi 15g, juhong 15g, Zhibanxia 15g, Zhikuandonghua 15g, Chaobaizhu 12g, Cishi 20g, Fangfeng 10g* | 1 dosage per day, intake twice a day |
| LU | 2017 | SLBZPAAS | Shen Ling Bai Zhu Powder Add And Subtract | *Danshen 20g, Dangshen 20g, Shanyao 30g, Yiyiren 20g, Lianzirou 15g, Baibiandou 15g, Fuling 15g, Baizhu 10g, Jiegeng 10g, Sharen 10g, Gancao 6g* | 1 dosage per day, intake twice a day |
| LI | 2017 | MSGP | Modified Shen Ge Powder | *Wuweizi 30g, Xiyangshen 45g, Gejie 1pair, Chuanbeimu 45g* | 1 dosage per day, intake three times a day |
| GUO | 2017 | YYQFD | Yang Yin Qing Fei Decoction | *Shengdihuang 15g, Chaobaishao 15g, Maidong 12g, Chuanbeimu 12g, Xuanshen 12g, Mudanpi 8g, Gancao 6g, Bohe 5g* | 1 dosage per day, intake twice a day |
| ZHAO | 2017 | SMLRD | Self-made Liu Ren Decoction | *Huomaren 10g, Yuliren 10g, Taoren 15g, Gualouren 10g, Xingren 10g, Boziren 10g, Jiegeng 10g, Baibu 10g, Zhiziyuan 10g, Fangfeng 10g, Huangqi 45g, Taizishen 15g, Baizhu 10g, Chenpi 10g, Tusizi 10g, Roucongrong 10g, Zhigancao 10g, Danggui 15g, Lugen 20g, Niubangzi 10g, Shanzha 10g* | 1 dosage per day, intake three times a day |
| LIN | 2017 | MLJZD | Modified Liu Jun Zi Decoction | *Dangshen 15g, Huangqi 15g, Fuling 15g, Hongjingtian 15g, Danshen 15g, Baizhu 10g, Fabanxia 10g, Xingren 10g, Zhike 10g, Tinglizi 10g, Chuanqiong 10g, Buguzhi 10g, Chenpi 6g, Gancao 5g* | 1 dosage per day, intake twice a day |
| LOU | 2016 | JQNSM | Jiang Qi Na Shen Method | *Suzi 12g, Qingbanxia 15g, Danggui 6g Qianhu 12g, Houpu 12g, Rougui 3g, Suye 9g, Zhigancao 6g* | 1 dosage per day, intake twice a day |
| YE | 2016 | TBFSM | Tiao Bu Fei Shen Method | *Dongchongxiacao 10g, Shanzhuyu 20g, Yinyanghuo 15g, Wuweizi 10g, Taizishen 30g, Baiguo 15g, Fuling 20g, Danshen 20g* | 1 dosage per day, intake twice a day |
| BIAN | 2016 | BSFCD | Bu Fei Fang Chuan Decoction | *Huangqi 30g, Ziyuan 10g, Renshen 10g, Wuweizi 5g, Jiegeng 10g, Baizhu 10g, Fangfeng 10g, Gejie 15g, Jiaogulan 15g* | 1 dosage per day, intake twice a day |
| LI | 2016 | ZLFSQXDGFTJ | Zhi Liao Fei Shen Qi Xu De Gui Fan Tang Ji | *Renshen 9g, Huangqi 15g, Goujizi 12g, Shanzhuyu 9g, Wuweizi 9g, Yinyanghuo 9g, Zhebeimu 9g, Zisuzi 9g, Chishao 12g, Dilong 12g, Chenpi 9g, Zhigancao 6g* | 1 dosage per day, intake twice a day |
| TANG | 2016 | ZCG | Zhi Chuan Granule | *Xianlingpi 9g, Bajitian 9g, Shechuangzi 3g* | 1 dosage per day, intake twice a day |
| GUO | 2016 | MZFWDF | Man Zu Fei Wen Ding Decoction | *Huangqi 15g, Baizhu 10g, Danggui 15g, Dangshen 15g, Bajitian 10g, Buguzhi 10g, Beishashen 10g, Nanshashen 10g* | 1 dosage per day, intake twice a day |
| FANG | 2016 | SLBZPAAS | Shen Ling Bai Zhu Powder Add And Substract | *Baibiandou 6g, Baizhu 12g, Fuling 12g, Gancao 3g, Jiegeng 6g, Lianzi 9g, Dangshen 20g, Sharen 10g, Shanyao 30g, Yiyiren 20g* | 1 dosage per day, intake twice a day |
| CHEN | 2016 | LJF | Li Jin Fomula | *Dangshen 10g, Huangqi 15g, Maidong 10g, Wuweizi 3g, Ziyuan 10g, Chuanbei 6g, Gejie 5g, Baizhu 10g, Fangfeng 6g, Fuling 10g, Chenpi 6g, Gancao 6g* | 1 dosage per day, intake twice a day |
| HU | 2016 | BFDAAS | Bai Fei Decoction Add And Subtract | *Taizishen 20g, Maidong 10g, Wuweizi 10g, Qingbanxia 9g, Chuanbeimu 10g, Gualou 10g, Danggui 10g, Chishao 10g* | 1 dosage per day, intake once a day |
| LIANG | 2015 | TBFSM | Tiao Bu Fei Shen Method | *Yinyanghuo 15g, Wuweizi 10g, Taizishen 30g, Baiguo 15g, Fuling 20g, Danshen 20g* | 1 dosage per day, intake twice a day |
| WANG | 2015 | BFYSD | Bu Fei Yi Shen Decoction | *Shanzhuyu 10g, Danggui 15g, Wuweizi 15g, Huangqi 30g, Danshen 10g, Zhigancao 6g, Fuling 18g, Shoudihuang 20g, Chenpi 10g, Chenxiang 5g, Rougui 3g, Dilong 10g, Gejie 2pcs, Baizhu 15g* | 1 dosage per day, intake twice a day |
| SI | 2015 | BFJPYSD | Bu Fei Jian Pi Yi Shen Decoction | *Huangqi 30g, Dangshen 20g, Chaobaizhu 20g, Fuling 20g, Shoudi 25g, Buguzhi 20g, Shanyao 20g, Shanzhuyu 15g, Danshen 20g, Chenpi 15g, Wuweizi 5g, Chuanbei 5g, Zhigancao 5g* | 1 dosage per day, intake twice a day |
| WANG | 2015 | BZGWHJ | Bu Zhong Gu Wei He Ji | *Huangqi 30g, Dangshen 15g, Baizhu 15g, Danggui 12g, Fangfeng 12g, Xingren 12g, Zhibanxia 9g, Shengma 9g, Chaihu 9g, Dilong 9g, Suzi 10g, Mahuang 3g, Chenpi 6g, Zhigancao 6g* | 1 dosage per day, intake twice a day |
| LI | 2015 | YQBSHXF | Yi QI Bu Shen Huo Xue Formula | *Huangqi 20g, Dangshen 20g, Shoudihuang 15g, Buguzhi 15, Wuweizi 15g, Huangjing 15g, Fabanxia 10g, Kuandonghua 15g, Ziyuan 15g, Zisuzi 15g, Yinyanghuo 15g, Sumu 10g, Shanzhuyurou 15g, Gancao 5g* | 1 dosage per day, intake twice a day |
| HE | 2015 | SLBZG | Shen Ling Bai Zhu Granule | *Renshen, Chaobaizhu, Fuling, Chaoyiyiren, Shanyao, Chaobaibiandou, Sharen, Lianzi, Jiegeng, Gancao* | 1 dosage per day, intake twice a day |
| HUANG | 2015 | KFZY | Kou Fu Zhong Yao | *Baizhu 15g, Chenpi 15g, Xisharen 15g, Yinyanghuo 20g, Yizhiren 20g, Shengjiang 50g, Zhigancao 5g* | 1 dosage per day, intake three times a day |
| ZHANG | 2015 | SXDAAS | Sheng Xian Decoction Add And Subtract | *Huangqi 30g, Zhimu 15g, Chaihu 15g, Jiegeng 12g, Shengma 10g* | 1 dosage per day, intake twice a day |
| LUO | 2014 | BFPCD | Bu Fei Ping Chuan Decoction | *Renshen 10g, Shenghuangqi 40g, Baizhu 10g, Fangfeng 6g, Fuling 12g, Maidong 10g, Yuxingcao 20g, Chenxiang 3g, Gejie 3g, Ziheche 9g, Danshen 15g, Chenpi 10g* | 1 dosage per day, intake three times a day |
| WEN | 2014 | BFDAAS | Bu Fei Decoction Add And Subtract | *Huangqi 30g, Dangshen 15g, Baibu 15g, Wuweizi 10g, Sangbaipi 10g, Danshen 10g* | 1 dosage per day, intake twice a day |
| LI | 2014 | BFNSF | Bu Fei Na Shen Formula | *Huangqi 30g, Gejie 2pcs, Baizhu 15g, Fuling 18g, Shoudihuang 20g, Shanzhuyu 10g, Danggui 15g, Wuweizi 15g, Chenpi 10g, Chenxiang 5g, Rougui 3g, Dilong 10g, Danshen 10g, Zhigancao 6g* | 1 dosage per day, intake twice a day |
| QI | 2014 | WSBFHTT | Wen Shen Bu Fei Hua Tan Therapy | *Shoudihuang 15g, Buguzhi 10g, Huangqi 15g, Taizishen 10g, Gejie 10g, Duzhong 10g, Tusizi 15g, Fabanxia 10g, Fuling 20g, Baizhu 15g, Ziyuan 15g, Kuandonghua 15g, Suzi 10g, Xingren 10g, Hezi 10g* | 1 dosage per day, intake twice a day |
| ZENG | 2014 | MSZYQD + GZLMD | Modified San Zi Yang Qin Decocton + Gui Zhi Long Mu Decoction | *Laifuzi 9g, Baijiezi 9g, Suzi 9g, Guizhi 15g, Gancao 30g, Longgu 30g, Muli 30g* | 1 dosage per day, intake twice a day |
| ZHANG | 2014 | QZYQDG | Quan Zhen Yi Qi Decoction Granule | *Shengshaishen 15g, Maidong 15g, Shoudihuang 15g, Danfuzi 6g, Baizhu 6g, Niuxi 15g, Wuweizi 6g* | 1 dosage per day, intake twice a day |
| CHEN | 2013 | BFYSQTD | Bu Fei Yi Shen Qu Tan Decoction | *Shenghuangqi 30g, Dangshen 12g, Jiaobaizhu 15g, Fuling 10g, Shoudihuang 10g, Buguzhi 12g, Wuweizi 10g, Chaosangbaipi 15g, Zhibaibu 10g, Zhiziyuan 10g, Chuanqiong 12g, Chaozhike 10g, Sharen 10g* | 1 dosage per day, intake twice a day |
| TAN | 2013 | BZYQM | Bu Zhong Yi Qi Method | *Dangshen 15g, Chaobaizhu 15g, Fuling 20g, Huangqi 30g, Danggui 15g, Chenpi 12g, Jiegeng 15g, Zhimahuang 10g, Shengma 3g, Chaihu 15g, Qingbanxia 9g, Gancao 6g* | 1 dosage per day, intake twice a day |
| ZHANG | 2013 | QYJDF | Qu Yu Jie Du Formula | *Chuanqiong 15g, Chishao 20g, Danggui 10g, Suzi 12g, Jinyinhua 15, Sigualuo 6g* | 1 dosage per day, intake twice a day |
| LIANG | 2013 | RFJPBSD | Run Fei Jian Pi Bu Shen Decoction | *Shashen 15g, Maidong 15g, Digupi 15g, Sangbaipi 15g, Xiyangshen 15g, Fuling 15g, Bajitian 15g, Guangdilong 15g, Baizhu 10g, Chuanqiong 10g, Muli 30g, Zisuzi 5g, Quanxie 5g, Ziheche 10g* | 1 dosage per day, intake twice a day |
| JIANG | 2013 | SLBZG | Shen Ling Bai Zhu Granule | *Renshen 6g, Chaobaizhu 6g, Fuling 6g, Chaoyiyiren 6g, Shanyao 6g, Chaobaibiandou 6g, Sharen 6g, Lianzi 6g, Jiegeng 6g, Gancao 6g* | 1 dosage per day, intake twice a day |
| WANG | 2013 | SLBZDAAS | Shen Ling Bai Zhu Granule Add And Subtract | *Dangshen 20g, Baizhu 10g, Fuling 15g, Chenpi 10g, Baibiandou 15g, Lianzirou 15g, Huaishanyao 30g, Jiegeng 10g, Yiyiren 20g, Sharen 10g, Danshen 20g, Gancao 6g* | 1 dosage per day, intake twice a day |
| DAI | 2013 | SMBPYQD | Self-made Bu Pi Yi Qi Decoction | *Dangshen 20g, Renshen 10g, Huangqi 20g, Shanyao 20g, Baizhu 15g, Fuling 15g, Wuweizi 10g, Shoudi 15g, Xingren 10g, Ziyuan 15g, Taoren 10g, Gancao 5g* | 1 dosage per day, intake twice a day |
| CHEN | 2012 | GBQTHYD | Gu Ben Yu Tan Hua Yu Decoction | *Shenhuangqi 15g, Baizhu 10g, Chenpi 6g, Dangshen 10g, Fangfeng 6g, Fuling 10g, Huaishanyao 10g, Shanyurou 6g, Fabanxia 6g, Huangjing 6g, Shenggancao 6g* | 1 dosage per day, intake twice a day |
| CHEN | 2011 | FGFZM | Fang Gan Fu Zheng Mixture | *Shenqi 30g, Dangshen 15g, Chaobaizhu 15g, Buguzhi 15g, Yinyanghuo 10g, Shanzhuyu 15g, Chenpi 12g, Fabanxia 9g, Fuling 20g, Xingren 10g, Taoren 10g, Honghua 15g, Danshen 20g, Chishao 10g, Dilong 10g, Danggui 10g, Jinyinteng 15g* | 1 dosage per day, intake twice a day |
| GONG | 2011 | SETGM | Strenthening Earth To Generate Metal | *Huangqi 45g, Wuzhualong 30g, Dangshen 30g, Chaihu 15g, Baizhu 15g, Danggui 10g, Gancao 6g* | 1 dosage per day, intake twice a day |
| TANG | 2010 | JPYSD | Jian Pi Yi Shen Decoction | *Huangqi 20g, Dangshen 10g, Baizhu 10g, Nanshashen 15g, Beishashen 15g, Buguzhi 10g, Bajitian 10g, Danggui 15g* | 1 dosage per day, intake twice a day |
| HE | 2010 | SETGM | Strenthening Earth To Generate Metal |  | 1 dosage per day, intake twice a day |
| WANG | 2009 | SMBFTFD | Self-made Bu Fei Tong Fu Decoction | *Zhihuangqi 30g, Dangshen 15g, Quangualou 20g, Xingren 6g, Zhidahuang 6g, Taoren 6g, Hetaoren 10g, Huaishanyao 10g, Suzi 10g, Houpu 9g, Wumei 3g* | 1 dosage per day, intake three times a day |
| WANG | 2008 | BFTFD | Bu Fei Tong fu Decoction | *Zhihuangqi 30g, DAngshen 15g, Shanyao 10g, Quangualou 20g, Xingren 6g, Taoren 6g, Huhetao 10g, Zisuzi 10g, Houpu 9g, Zhidahuang 6g, Wumei 3g* | 1 dosage per day, intake three times a day |
| JIANG | 2008 | SMYQHXD | Self-made Yi Qi Huo Xue Decoction | *Huangqi 30g, Dilong 6g, Xuanshen 15g, Danshen 15g, Taizishen 15g, Danggui 15g* | 1 dosage per day, intake three times a day |
| HONG | 2008 | YFN | Yu Fei Ning | *Dangshen 7.5g, Huangqi 7.5g, Baizhu 5g, Fangfeng 5g, Huangjing 5g, Shanzhuyu 5g, Wuweizi 3g, Hetaorou 7.5g, Tusizi 5g, Baijitian 5g, Gualou 5g, Fabanxia 5g, Zhemeibu 5g, Danshen 5g, Taoren 5g* | 1 dosage per day, intake twice a day |

**Appendix 7. Characteristics of excluded studies table.**

| **PICOS category** | **Population** | **Interventions** | **Comparison** | **Outcomes** | **Study design** | **Total** |
| --- | --- | --- | --- | --- | --- | --- |
| **Number** | **64** | **97** | **9** | **12** | **41** | **223** |
| **Study** | **Reason for exclusion** | |  |  |  |  |

| ZHANG | 2019 | Study design: not RCT |
| --- | --- | --- |
| ZHANG | 2019 | Population: not stable COPD |
| PENG | 2019 | Population: not stable COPD |
| LI | 2019 | Study design: not RCT |
| LI | 2019 | Study design: not RCT |
| TONG | 2019 | Interventions: not decoction or granules |
| GUAN | 2019 | Study design: not RCT |
| WU | 2019 | Interventions: not decoction or granules |
| DENG | 2019 | Outcomes: not ITT analysis |
| ZHAO | 2019 | Interventions: not combined with CT |
| XUE | 2019 | Study design: not RCT |
| LAN | 2019 | Population: not stable COPD |
| LYU | 2019 | Interventions: not combined with CT |
| YANG | 2019 | Interventions: not decoction or granules |
| YU | 2019 | Study design: not RCT |
| WANG | 2019 | Outcomes: not ITT analysis |
| CHEN | 2019 | Population: not stable COPD |
| SHANG | 2019 | Interventions: not decoction or granules |
| WANG | 2019 | Population: not stable COPD |
| YU | 2019 | Population: not stable COPD |
| XIE | 2019 | Interventions: not decoction or granules |
| YIN | 2019 | Interventions: not decoction or granules |
| FAN | 2019 | Outcomes: not ITT analysis |
| HAN | 2019 | Study design: not RCT |
| FANG | 2018 | Interventions: not decoction or granules |
| LIU | 2018 | Population: not stable COPD |
| LI | 2018 | Population: not stable COPD |
| LIANG | 2018 | Study design: not RCT |
| ZHANG | 2018 | Interventions: not combined with CT |
| YANG | 2018 | Population: not stable COPD |
| ZHEN | 2018 | Interventions: not combined with CT |
| MAO | 2018 | Interventions: not decoction or granules |
| LIU | 2018 | Population: stable COPD with depression |
| CHEN | 2018 | Population: not stable COPD |
| JIANG | 2018 | Interventions: not combined with CT |
| GONG | 2018 | Population: not stable COPD |
| DENG | 2018 | Population: COPD staging too narrow |
| LIU | 2018 | Population: not stable COPD |
| JING | 2018 | Interventions: not decoction or granules |
| GAO | 2018 | Interventions: not combined with CT |
| WANG | 2018 | Population: not stable COPD |
| GUO | 2018 | Population: not stable COPD |
| YANG | 2018 | Interventions: not decoction or granules |
| ZHAO | 2018 | Study design: not RCT |
| WANG | 2018 | Interventions: not decoction or granules |
| WANG | 2018 | Population: not stable COPD |
| ZHONG | 2018 | Interventions: not decoction or granules |
| GUO | 2018 | Population: not stable COPD |
| QU | 2018 | Interventions: not combined with CT |
| GU | 2018 | Population: not stable COPD |
| HU | 2017 | Study design: not RCT |
| PI | 2017 | Interventions: not combined with CT |
| CHEN | 2017 | Population: not stable COPD |
| SUN | 2017 | Interventions: not decoction or granules |
| XIANG | 2017 | Interventions: not decoction or granules |
| FU | 2017 | Interventions: not decoction or granules |
| LI | 2017 | Interventions: not decoction or granules |
| KE | 2017 | Interventions: not combined with CT |
| KE | 2017 | Study design: not RCT |
| HUANG | 2017 | Population: not stable COPD |
| WEI | 2017 | Interventions: not decoction or granules |
| WANG | 2017 | Study design: not RCT |
| WEI | 2017 | Population: not stable COPD |
| ZHOU | 2017 | Population: not stable COPD |
| GAO | 2017 | Population: not stable COPD |
| SU | 2017 | Interventions: not decoction or granules |
| LI | 2017 | Interventions: not combined with CT |
| WANG | 2017 | Interventions: not decoction or granules |
| YANG | 2017 | Outcomes: not ITT analysis |
| ZHANG | 2017 | Study design: not RCT |
| DING | 2016 | Interventions: not combined with CT |
| LIU | 2016 | Outcomes: no primary and secondary outcome reported |
| WANG | 2016 | Interventions: not combined with CT |
| HUANG | 2016 | Study design: not RCT |
| SHEN | 2016 | Population: not stable COPD |
| LIU | 2016 | Population: not stable COPD |
| LI | 2016 | Comparison: no control group |
| YANG | 2016 | Interventions: not decoction or granules |
| XU | 2016 | Population: not stable COPD |
| CHEN | 2016 | Interventions: not decoction or granules |
| HUANG | 2016 | Study design: not RCT |
| CHU | 2016 | Study design: not RCT |
| CHEN | 2016 | Interventions: not decoction or granules |
| HE | 2016 | Population: not stable COPD |
| FENG | 2016 | Interventions: not combined with CT |
| JIAN | 2016 | Outcomes: no primary and secondary outcome reported |
| CHU | 2016 | Outcomes: no primary and secondary outcome reported |
| LU | 2016 | Interventions: not combined with CT |
| LI | 2016 | Study design: not RCT |
| WANG | 2016 | Interventions: not decoction or granules |
| CUI | 2015 | Interventions: not decoction or granules |
| LI | 2015 | Interventions: not decoction or granules |
| SONG | 2015 | Interventions: not combined with CT |
| HU | 2015 | Population: not stable COPD |
| KUANG | 2015 | Population: not stable COPD |
| CHENG | 2015 | Comparison: no control group |
| LI | 2015 | Interventions: not decoction or granules |
| LIU | 2015 | Population: not stable COPD |
| ZHANG | 2015 | Population: not stable COPD |
| XUE | 2015 | Population: not stable COPD |
| WANG | 2015 | Outcomes: not ITT analysis |
| DU | 2015 | Interventions: not decoction or granules |
| YAN | 2015 | Population: not stable COPD |
| HONG | 2015 | Outcomes: no primary and secondary outcome reported |
| AI | 2015 | Population: not stable COPD |
| HU | 2015 | Population: not stable COPD |
| CHEN | 2015 | Interventions: not decoction or granules |
| ZHANG | 2015 | Interventions: not decoction or granules |
| JIANG | 2015 | Interventions: not decoction or granules |
| TAN | 2015 | Interventions: not combined with CT |
| XIONG | 2015 | Population: not stable COPD |
| YI | 2015 | Population: not stable COPD |
| HUI | 2014 | Interventions: not decoction or granules |
| ZHAO | 2014 | Interventions: not combined with CT |
| SONG | 2014 | Population: not stable COPD |
| YANG | 2014 | Interventions: not combined with CT |
| WU | 2014 | Interventions: not decoction or granules |
| LIU | 2014 | Study design: not RCT |
| PENG | 2014 | Interventions: not combined with CT |
| LIU | 2014 | Population: not stable COPD |
| CHEN | 2014 | Interventions: not combined with CT |
| LING | 2014 | Population: not stable COPD |
| CHEN | 2014 | Population: not stable COPD |
| ZHU | 2014 | Population: not stable COPD |
| PEI | 2014 | Population: not stable COPD |
| WANG | 2014 | Population: not stable COPD |
| SU | 2014 | Population: not stable COPD |
| XUE | 2014 | Population: not stable COPD |
| HUANG | 2014 | Comparison: no control group |
| ZHAO | 2013 | Population: not stable COPD |
| ZHAO | 2013 | Population: not stable COPD |
| FU | 2013 | Interventions: not decoction or granules |
| LIU | 2013 | Study design: not RCT |
| WANG | 2013 | Population: not stable COPD |
| WU | 2013 | Interventions: not combined with CT |
| MENG | 2013 | Study design: not RCT |
| WANG | 2013 | Interventions: not combined with CT |
| LIU | 2013 | Study design: not RCT |
| JIN | 2012 | Study design: not RCT |
| SHI | 2012 | Interventions: more than CHM treatment |
| LI | 2012 | Study design: not RCT |
| LI | 2012 | Interventions: not combined with CT |
| ZHU | 2012 | Interventions: not combined with CT |
| HU | 2012 | Interventions: not decoction or granules |
| LU | 2012 | Population: not stable COPD |
| GUO | 2012 | Population: not stable COPD |
| LIANG | 2012 | Study design: not RCT |
| YANG | 2012 | Interventions: not combined with CT |
| MAO | 2012 | Population: not stable COPD |
| WANG | 2012 | Outcomes: not ITT analysis |
| XU | 2012 | Interventions: not decoction or granules |
| XIE | 2012 | Study design: not RCT |
| ZHENG | 2011 | Study design: not RCT |
| WANG | 2011 | Interventions: not decoction or granules |
| ZHOU | 2011 | Interventions: not combined with CT |
| ZHU | 2011 | Interventions: not combined with CT |
| HE | 2011 | Interventions: not combined with CT |
| ZHENG | 2011 | Interventions: not combined with CT |
| FANG | 2011 | Interventions: not decoction or granules |
| YU | 2011 | Interventions: not decoction or granules |
| YANG | 2011 | Interventions: more than CHM treatment |
| LIAO | 2011 | Study design: not RCT |
| HAN | 2011 | Interventions: not decoction or granules |
| CAO | 2011 | Interventions: not combined with CT |
| YANG | 2011 | Interventions: not CHM |
| JIANG | 2010 | Population: not stable COPD |
| FENG | 2010 | Study design: not ITT analysis |
| DAI | 2010 | Population: not stable COPD |
| LI | 2010 | Interventions: not decoction or granules |
| WANG | 2010 | Population: not stable COPD |
| LING | 2010 | Study design: not RCT |
| WANG | 2010 | Study design: not RCT |
| ZHANG | 2010 | Study design: not RCT |
| WANG | 2010 | Outcomes: no primary and secondary outcome reported |
| WANG | 2010 | Population: not stable COPD |
| LI | 2010 | Study design: not RCT |
| ZHUO | 2010 | Interventions: not decoction or granules |
| SUN | 2009 | Population: not stable COPD |
| MOU | 2009 | Interventions: not decoction or granules |
| LIU | 2009 | Interventions: not decoction or granules |
| FU | 2009 | Interventions: not combined with CT |
| GUO | 2008 | Interventions: not decoction or granules |
| ZHANG | 2008 | Population: not stable COPD |
| SHI | 2008 | Interventions: not combined with CT |
| NI | 2008 | Study design: not RCT |
| LIANG | 2008 | Population: not stable COPD |
| LI | 2008 | Interventions: not combined with CT |
| YAN | 2007 | Population: not stable COPD |
| ZHANG | 2007 | Interventions: not combined with CT |
| CHEN | 2007 | Interventions: not combined with CT |
| GAO | 2007 | Interventions: not decoction or granules |
| GAO | 2007 | Study design: not RCT |
| PENG | 2007 | Study design: not RCT |
| HUANG | 2005 | Interventions: not decoction or granules |
| HONG | 2005 | Interventions: not combined with CT |
| FENG | 2005 | Interventions: not decoction or granules |
| WU | 2004 | Interventions: not decoction or granules |
| HE | 2004 | Study design: not RCT |
| ZHANG | 2003 | Interventions: not decoction or granules |
| PAN | --- | Study design: not RCT |
| LYU | 2019 | Comparison: no control group |
| YU | 2019 | Comparison: no control group |
| YANG | 2018 | Interventions: not combined with CT |
| SUN | 2018 | Interventions: not combined with CT |
| LIU | 2018 | Population: not stable COPD |
| CHEN | 2018 | Interventions: not combined with CT |
| YAO | 2017 | Interventions: not combined with decoction or granules |
| LU | 2016 | Study design: not RCT |
| ZHANG | 2015 | Interventions: not decoction or granules, or acupuncture |
| WANG | 2014 | Population: not stable COPD |
| FAN | 2011 | Interventions: not combined with CT |
| YANG | 2010 | Comparison: no CT control group |
| WAN | 2007 | Comparison: no CT control group |
| SHERGIS | 2019 | Population: not stable COPD |
| CHUANG | 2015 | Interventions: not combined with decoction or granules |
| WANG | 2014 | Comparison: no CT control group |
| GUO | 2014 | Comparison: no CT control group |
| XIE | 2013 | Interventions: not combined with CT |
| LI | 2012 | Interventions: not combined with CT |
| CURCIO | 2007 | Outcomes: no primary and secondary outcome reported |
| FENG | 2016 | Interventions: not combined with CT |
| SUZUKI | 2012 | Comparison: no CT control group |
| SUZUKI | 2008 | Study design: not RCT |
| JOBST | 1986 | Interventions: not combined with CT |
| CT: convention treatment, RCT: randomized controlled trial, COPD: Chronic obstructive pulmonary disease, ITT: intention-to-treat | | |
